# Macroalgae as a Source of Valuable Antimicrobial Compounds: Extraction and Applications

**DOI:** 10.3390/antibiotics9100642

**Published:** 2020-09-25

**Authors:** Aurora Silva, Sofia A. Silva, M. Carpena, P. Garcia-Oliveira, P. Gullón, M. Fátima Barroso, M.A. Prieto, J. Simal-Gandara

**Affiliations:** 1 Nutrition and Bromatology Group, Department of Analytical and Food Chemistry, Faculty of Food Science and Technology, University of Vigo, Ourense Campus, E32004 Ourense, Spain; mass@isep.ipp.pt (A.S.); maria.carpena.rodriguez@uvigo.es (M.C.); paula.garcia.oliveira@uvigo.es (P.G.-O.); patrigullon@gmail.com (P.G.); 2 REQUIMTE/LAQV, Instituto Superior de Engenharia do Porto, Instituto Politécnico do Porto, Rua Dr António Bernardino de Almeida 431, 4200-072 Porto, Portugal; MFSBA@isep.ipp.pt; 3 Departamento de Química, Universidade de Aveiro, 3810-168 Aveiro, Portugal; sofia.silva96@gmail.com; 4 Centro de Investigação de Montanha (CIMO), Instituto Politécnico de Bragança, Campus de Santa Apolonia, 5300-253 Bragança, Portugal

**Keywords:** antimicrobial applications, antimicrobial compounds, bioactive compounds, macroalgae, novel technologies

## Abstract

In the last few decades, attention on new natural antimicrobial compounds has arisen due to a change in consumer preferences and the increase in the number of resistant microorganisms. Macroalgae play a special role in the pursuit of new active molecules as they have been traditionally consumed and are known for their chemical and nutritional composition and their biological properties, including antimicrobial activity. Among the bioactive molecules of algae, proteins and peptides, polysaccharides, polyphenols, polyunsaturated fatty acids and pigments can be highlighted. However, for the complete obtaining and incorporation of these molecules, it is essential to achieve easy, profitable and sustainable recovery of these compounds. For this purpose, novel liquid–liquid and solid–liquid extraction techniques have been studied, such as supercritical, ultrasound, microwave, enzymatic, high pressure, accelerated solvent and intensity pulsed electric fields extraction techniques. Moreover, different applications have been proposed for these compounds, such as preservatives in the food or cosmetic industries, as antibiotics in the pharmaceutical industry, as antibiofilm, antifouling, coating in active packaging, prebiotics or in nanoparticles. This review presents the main antimicrobial potential of macroalgae, their specific bioactive compounds and novel green extraction technologies to efficiently extract them, with emphasis on the antibacterial and antifungal data and their applications.

## 1. Introduction 

Approximately 70% of the Earth’s surface is covered by marine waterand thus, the marine world is home to a huge diversity of species. Several organisms have been proposed as sources of known beneficial compounds and other new molecules with biological potential [1,2]. Nowadays, there are more than 200,000 eukaryotic marine species validated, among which, algae contribute nearly 44,000 described species [3]. Among algae, macroalgae (also called seaweed) constitute a new source of compounds, as they have been used traditionally for nutritional or medicinal purposes [4]. They are defined as marine macroscopic eukaryote photosynthetic organisms. Among them, plenty of divisions can be established depending on the chosen criteria, however the most common classification divides macroalgae in three groups depending on their pigments: green (Chlorophyceae), red (Rhodophyceae) and brown algae (Ochrophyta) [5,6].

More recently, functional products and especially, natural functional ingredients have enjoyed a boost in consumer demand. These products are usually preferred by the client over synthetic ingredients; a trend that is growing not only in the food industry but also in other sectors. In this context, macroalgae entail a source of valuable compounds for their nutritional and chemical composition [7]. Algae’s nutritional profile usually consists of minerals (7–36%), lipids (1–5%), polysaccharides (15–76%) and proteins (5–47%) [3,8,9]. Concretely, algae polysaccharides (namely, agar, alginate or carrageenans, among others) have been widely studied for their food applications as thickener, stabilizer or emulsifier agents [10,11]. On the other hand, even though macroalgae have a low lipid content, they have a high proportion of poly-unsaturated fatty acids (PUFAs) and other lipid compounds with beneficial health properties [9,12]. Moreover, they also show an elevated content of micro-nutrients such as vitamins and other secondary metabolites, usually antioxidants, such as polyphenols or pigments [10]. In sight of the variety of active molecules reported in algae, their extracts have been submitted to different bioactivity tests showing plenty of biological properties, such as: anti-inflammatory, antioxidant, antimicrobial, antidiabetic, anticancer, neuroprotective and photoprotective, among others [13,14,15]. Regarding all these aspects, macroalgae may be considered as a source of active molecules with biological properties and with a huge potential for application in food, cosmetic and pharmacological industries, not only because of their composition but also for their diversity and the availability of resources [16,17,18].

During the last decades, two main trends have stimulated interest in new natural antimicrobial compounds. First, natural ingredients with preservative properties have experienced an increasing demand, in replacement of the use of synthetic ingredients, to prevent microbial contamination as they are safer, ecofriendly, they possess a wide spectra of actions and they avoid some of the side-effects associated with synthetic antimicrobials [19,20]. Second, in recent decades, an increase in the number of pathogens (bacteria and fungi) resistant to antimicrobial drugs has occurred. This issue is considered now as a public health problem since traditional antibiotics and antifungals have lost efficacy [21].

**Figure 1:**
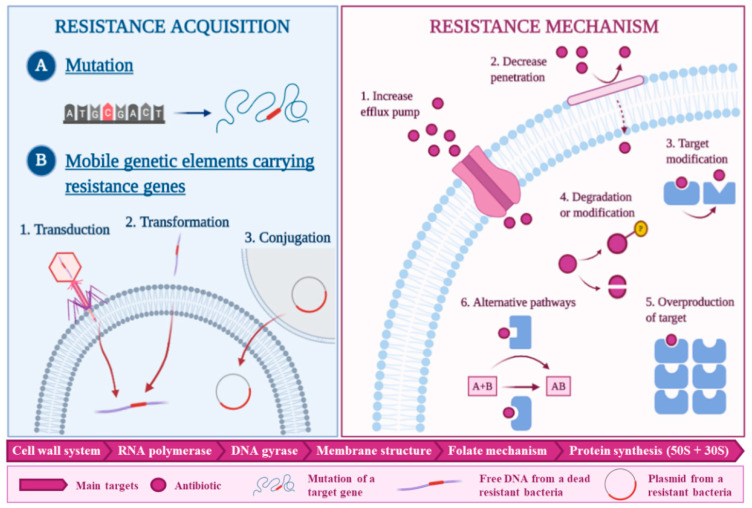
Summary of the resistance acquisition pathways: Mutation and mobile genetic elements: By transduction, transformation or conjugation. Schema of the six main mechanisms of antibiotics resistance. Modified from [1,26,27].

In Figure 1, a schematic summary of the main resistance acquisition pathways (fundamentally by mutation or by acquiring mobile genetic elements with resistance genes) [22] and the main mechanisms of resistance to antibiotics is shown. In this context, marine macroalgae have shown antimicrobial potential and in some cases, a synergistic effect with conventional antimicrobial agents against drug-resistant pathogens [1]. Thus, this association can be applied to the pharmaceutical sector and also to the food industry, where consumer resistance is also a reality and food-spoilage-microorganisms control is a must in the food chain supply [23]. Nevertheless, the search for antimicrobial compounds in algae is not a recent idea. A study carried out in 1974, screened 151 species of British marine algae against different microorganisms in order to find new alternatives for the production of antibiotics [24]. However, considering the reviewed bibliography, the vast majority of the studies have focused on the general screening of the antimicrobial properties of algae extracts, whereas information about the purified molecule’s specific mechanism of action is quite scarce, with polyphenols being the molecules most studied [20,23]. Moreover, research has mostly focused on clinical bacteria, not on food related pathogens [25].

Given the actual interest in identifying antimicrobial compounds from algae, it is essential to achieve profitable and sustainable recovery of these compounds, in an easy and fast process [28]. Sometimes these compounds could be synthesized chemically, however, regarding the availability of algae, their recovery using green extraction technologies is an economic and environmentally friendly alternative that also avoids the use of dangerous chemical compounds. The extraction of bioactive compounds has always been a fundamental step in recovering these molecules from vegetal matrixes. New techniques have been developed in order to reduce extraction time, energy consumption, quantity of solvent, environmental implications, economical cost and waste productions while increasing the extraction efficiency and the quality of the obtained extract [29]. 

The most common classic technique applied to extract bioactive compounds from macroalgae is the maceration at different temperatures, followed by Soxhlet. It is also notable that the most frequently chosen solvents are methanol and ethanol even though other solvents with different characteristics are also successfully used. In this regard, Table 1 presents a compilation of the last 10 years of literature, refereeing studied algae species, the focus on the extraction conditions and highlighting the major outcomes achieved. Nowadays, novel liquid–liquid and solid–liquid extraction techniques are currently more applied, including: supercritical, ultrasound, microwave, enzymatic, high pressure, accelerated solvent and intensity pulsed electric fields extraction techniques [30]. In this regard, the combination of green technologies and safe environmental solvents is desirable to obtain efficient extraction of biocompounds, preserving their biological properties and opening the door to their implementation in the food, cosmetic and pharmaceutical industries. Once the target compounds are efficiently well extracted, the resultant extracts can be incorporated into different products. In general, the most common application would be the incorporation into food matrixes as preservatives [25], to cosmetic products [31] or as antibiotics with a synergistic effect on the pharmaceutical industry [1]. Moreover, other applications have been investigated such as antibiofilm [32], antifouling [33], coating in active packaging [34] or as prebiotics [11], among others. Therefore, the aim of this article is to review the main compounds responsible for the antimicrobial activity of algae, the novel extraction techniques for obtaining them and their applications. 

## 2. Macroalgae as a Promising Source of Valuable Antimicrobial Compounds 

The nutritional composition of seaweed is strongly influenced by factors such as the species, environmental conditions, geographical place, seasonality and characteristics of the growth medium as well as by the developmental stage [97]. Seaweeds are an excellent source of biologically active compounds that includes proteins and peptides, polysaccharides, polyphenols, PUFAs and pigments [98,99]. The resistance of pathogenic microorganisms to synthetic antibiotics has become a concern of public health systems [33] and therefore, it is imperative to explore new alternatives to solve this problem. In particular, among the different compounds that exhibit bioactivity that are present in macroalgae, interest in their antimicrobial potential has increased in the last few years in order to develop new antimicrobial therapies with less secondary effects, that are more cost effective and with minor toxicity, when compared to the synthetic antibiotics [33]. Although research on the antimicrobial properties of seaweed compounds is a topic of great interest, until now the attribution of a particular compound to such activity was a challenge as they are usually evaluated as extracts and not as a compound constituted by different biomolecules; in most cases, the antimicrobial effect is probably a consequence of a synergic effect between these compounds. The main components of seaweeds are described in the following sections. 

### 2.1. Protein and Peptides

The protein content is highly variable, ranging between 10 to 30% of the dry weight (DW) in red seaweeds, from 5 to 15% DW in brown seaweeds and from 3 to 47% DW in green seaweeds [9,97]. Moreover, these contents vary depending on the season, founding the highest concentration during the winter–early spring and the lowest during summer–early autumn [97,100,101]. The proteins that are actively functional in seaweeds belong to two groups, namely, lectins and phycobiliproteins [14,102]. Particularly, whereas lectins have been identified in several species of seaweeds, phycobiliproteins are found in red macroalgae [102].

Protein from seaweeds contains all amino acids, chiefly glycine, alanine, arginine, proline, glutamic and aspartic acids [103]. However, seaweeds present reduced content of lysine, threonine, tryptophan, cysteine and methionine in comparison with other protein foods [102]. Peptides obtained from seaweeds have experienced an increasing interest in recent years due to their multiple bioactivity compounds (angiotensin converting enzyme inhibition, antihypertensive, antioxidative and antidiabetic) [104].

Regarding the antimicrobial activity, the only seaweed protein described in the literature as antibacterial is lectin [14,105]. The information about bioactive peptides with antibacterial properties is scarce. For example, Beaulieu et al. (2015) obtained antibacterial peptides after hydrolysis of proteins from macroalgae *S. longicruris*. These authors evaluated the antibacterial activity of the >10 kDa protein hydrolysate fraction against *S. aureus* and observed a significant decrease in the maximum specific growth rate when using a quantity between 0.31 mg/mL to 2.5 mg/mL [105]. Another example is the study by Cordeiro et al. (2006), which extracted protein fractions rich in lectin from the red seaweed *H. musciformis* and assessed its antifungal properties against human pathogen yeasts *C. albican*s and *C. guilliermondii*. Specifically, the F40/70 fraction demonstrated the capacity to inhibit the growth of *C. guilliermondii* at 45, 100 and 450 μg protein/mL, but a poor inhibition was observed against *C. albicans* independent of the evaluated concentrations [106]. Lectin was also isolated from red macroalgae *S. filiformis* [107]. Antibacterial activity was assessed on the growth of eight Gram-negative (*E. coli*, *S. marcescens*, *S. typhi*, *S. typhimurium*, *K. pneumoniae*, *E. aerogenes*, *Proteus* spp, and *Pseudomonas aeruginosa*) and three Gram-positive (*B. cereus*, *B. subtilis* and *S. aureus*). The authors found different results regarding the effects of lectin against Gram-negative and Gram-positive bacteria growth. The lectin at 500 μg/mL stimulated the growth of *B. c*ereus and inhibited the growth of *S. marcescens*, *S. typhi*, *K. pneumoniae*, *E. aerogenes*, *Proteus* sp, and *P. aeruginosa* at 1000 μg/mL. The compound did not exhibit an effect, at any of the tested concentrations, on the growth of *S. aureus* and *B. subtilis*, or on *E. coli* and *S. typhimurium*. The authors concluded that more studies are necessary to evaluate the action of lectin on bacterial growth in order to evaluate further clinical applications.

### 2.2. Polysaccharides

Polysaccharides are the main components of seaweeds, having a structural role in algal cell walls [108]. Polysaccharides can be neutral or acidic, linear or branched [108]. Green, red and brown algae are defined by the presence of sulfated polysaccharides with a high degree of complexity in the range from 4% to 76% DW [109]. In green seaweeds, polysaccharides represent between 38 and 54% of their dry matter [110]. These macroalgae are rich in ulvan (another sulfate polysaccharide), sulfated rhamnans and sulfated galactans. The main component of these polysaccharides are sulfate groups and moreover they contain repeating units of rhamnose, xylose and uronic acids (glucuronic and iduronic acids) and minor contents of glucose, galactose, rhamnose and arabinose [110]. Red seaweeds present different sulfated galactans, sulfated rhamnans or mannans, carrageenans and agars. The main polysaccharides found in red macroalgae are galactans with a backbone composed of repeating units of three-linked β-D-galactopyranosyl and four-linked α-galactopyranosyl units: if the configuration of the four-linked α-galactopyranosyl units is L the polysaccharide is agar and if the configuration of the four-linked α-galactopyranosyl units is D then the polysaccharide is carrageenan. Agars are typically low in sulfate ester substitution, whereas carrageenans are comparatively rich in sulfate ester substitution [111]. Brown macroalgae are a rich source of alginate with a content that varies between 14 and 40% of their dry mass [7]. After the alginates, β-glucans (laminarans), cellulose and heteroglycans are present in important amounts in brown algae [7]. Laminarans are β-D-glucans with low molecular weight that are linear polysaccharides of glucose linked by 1→3 β-glycosidic bonds. There are different types of laminarans classed in accordance of their length and branching degree. Laminarans contain some 6-O-branches in their backbone and some β-(1→6)-intrachain links. Fucoidans are a family of sulfated homo and heteropolysaccharides that are mainly composed of (1→3)-linked-L-fucopyranose units. Moreover, fucoidans can present a main chain of alternating (1→3) and (1→4)-linked-L-fucopyranose units, and sulfate groups located mainly at C2, C4, or disubstituted at both C2 and C4. In addition, acetyl groups and D-galactose, D-xylose, D-mannose, L-rhamnose and D-glucuronic acid residues were identified as components of fucoidans [112].

The antimicrobial activity of polysaccharides from seaweeds depends on factors such as molecular weight, charge density, sulfated content in the case of sulfated polysaccharides and structural and conformation characteristics [33]. Several studies have been reported. For example, sulphated polysaccharides (alginates, fucoidans and laminaran) extracted from different seaweeds, such as *L. japonica*, *A. nodosum*, or *U. pinnafitida* have demonstrated an inhibitory effect on the growth of pathogenic bacteria [113]. Similarly, extracts rich in either laminarin or fucoidan isolated from *Laminaria* spp, decreased the fecal *E. coli* populations in piglets (0.3 and 0.24 g/kg, respectively), observing a reduction in the initial bacterial load in derived raw meat products [114].

### 2.3. Fatty Acids 

Seaweeds produce PUFAs and are also important sources of essential fatty acids [115,116,117]. The most predominant PUFAs are ω3 such as 16:4 ω3 and 18:4 ω3 and some species also present important amounts of eicosapentaenoic acid (20:5 ω3, EPA) [118], α-linolenic (18:3 ω-3), octadecatetraenoic (18:4ω-3) and arachidonic (20:4ω-6) [116]. Several studies have reported the antimicrobial properties of macroalgal fatty acids. For example, different sulfolipid classes isolated from the total lipids of two species of *U. fasciata (*Chlorophyta), *L. papillosa*, *G. cylindriea (*Rhodophyta), *D. fasciola* and *T. atomaria (*Ochrophyta). Authors assessed the antimicrobial activity of these compounds and they observed a high inhibition of the growth of *B. subtilis* and *E. coli,* using 100 μg/well. However, these compounds did not exhibit any inhibition against the fungi or yeast tested [119]. Another study obtained *G. vermiculophylla*, *P. dioica* and *C. crispus* using solvents with different polarity (DEt < EtOAc < MeOH:H_2_O (1:1)). The authors identified the fatty acid profile of ethyl acetate extracts and observed that saturated fatty acids (SFA), especially palmitic acid (16:0) was the more abundant, followed by polyunsaturated fatty acids (PUFA) and monounsaturated fatty acids (MUFA). They tested the antimicrobial activity against Gram-positive bacteria (*L. innocua*, *B. cereus*, *E. faecalis*, *L. brevis*, *S. aureus* and Gram negative *(E. coli*, *S. enteritidis*, *P. aeruginosa)* and the yeast *Candida* sp. The EtOAc extract showed the high inhibition capacity of the tested strains [17].

### 2.4. Polyphenolic Compounds 

Polyphenolic compounds are secondary metabolites of seaweeds whose composition vary from simple phenolic acids to complex molecules such as phlorotannins [120]. Macroalgae are excellent sources of catechins, flavonols and phlorotannins [121,122]. Green and red algae contain high proportions of bromophenols, phenolic acids and flavonoids. Brown algae present predominant polyphenolic compounds such us phlorotannins, which are complex polymers of phloroglucinol (1,3,5-trihydroxybenzene), and are classified into different groups in function of the bonds of the phloroglucinol units. They are divided in eckols and carmalols (dibenzodioxin linkage), fuhalols (ether bonds and hydroxyl groups), fucophlorethols (ether and phenyl bonds), phlorethols (ether bonds), fucols (phanyl linkages), and ishofuhalols [121,123]. In addition, some brown algae can contain bromo-, chloro- and iodo-phlorotannins [123]. These compounds represent 20% DW of algae [120] and have only been described in the composition of brown algae [123]. Other polyphenols such as catechins, flavonoids and flavonol glycosides have been identified in brown seaweeds [124].

Referring to the antimicrobial activity of polyphenols, several authors have studied the properties of phlorotanins. In this context, Nagayama et al. (2002) [125] evaluated the antibacterial effect of phlorotannins from *E. kurome* against several food-borne pathogenic bacteria, different MRSA strains and *S. pyogenes*. They observed that the phlorotannins were effective against MRSA and also *Campylobacter* spp., which presented the highest susceptibility to these compounds. The minimal bactericidal concentration of the crude phlorotannins, dieckol and 8,8′-bieckol against *C. jejuni* were 50 mg/L, 0.03 μmol/mL and 0.03 μmol/mL, respectively [125].

### 2.5. Pigments

Based on their content in pigments, macroalgae are classified into three groups: green algae (Chlorophyta, ca. 1200 species), red algae (Rhodophyta, ca. 6000 species) and brown algae (Ochrophyta, ca. 1750 species). Macroalgae are described to present three types of natural pigments: chlorophylls, carotenoids and phycobilins. Chlorophylls are greenish lipid-soluble natural pigments which contain a porphyrin ring and are found in all algae [102]. Four forms of chlorophylls have been identified in macroalgae with the most important being chlorophyll a; chlorophyll b and c are also described and the chlorophyll d is present in red algae [102]. Phycobiliproteins are other natural pigments and are water soluble fluorescent proteins present in seaweeds [126,127]. There are three types of phycobiliproteins: phycocyanins (blue pigment), phycoerytrins (red pigment) and allophycocyanins (light-blue pigment) [102,126], with phycoerytrins being the most abundant in many red macroalgae species. Carotenoids are linear polyenes and can be classified in carotenes (α y β-carotene, lycopene) and xanthophylls (fucoxanthin, violaxanthin, antheraxanthin, zeaxanthin, lutein, neoxanthin). The most abundant carotenoid is fucoxanthin, a brown pigment that confers the coloration to brown algae [102].

Regarding the antimicrobial activity of pigments, there are few studies that evaluate the activity of isolated compounds. A recent study has evaluated the antimicrobial properties of fucoxanthin against different Gram-positive and Gram-negative bacteria. The results showed that the compound was more effective against Gram-positive bacteria, with *S. agalactiae* being the most affected bacteria (with a MIC of 62.5 µg/mL), followed by *S. epidermidis* and *S. aureus* [128]. 

## 3. Mechanisms of Action of Antimicrobial Compounds 

**Figure 2:**
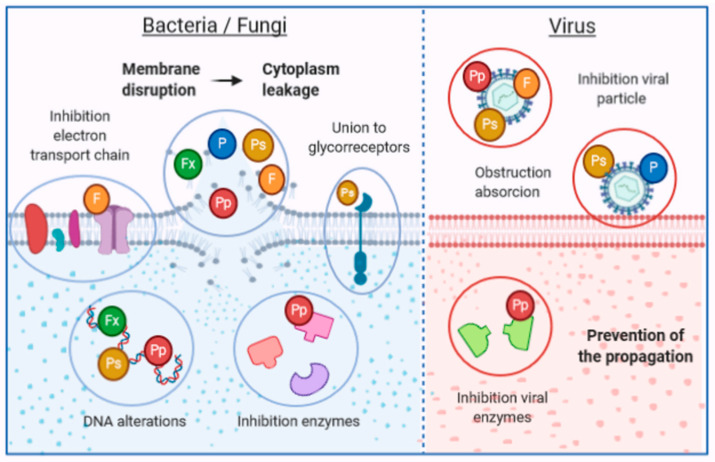
Schematic illustration of the main action mechanisms of antimicrobial compounds extracted from macroalgae species.

In the previous section, numerous compounds extracted from macroalgae with proven antimicrobial activity are mentioned. In the following paragraphs, the mechanisms of action of these compounds will be explained. A summary of these mechanisms is presented in (Figure 2). In Table 2, several studies demonstrating the antimicrobial properties of macroalgae compounds have been compiled. 

In the case of proteins and peptides, their inhibitory effects are associated with their amphiphilic nature, which allows them to interact with polar and non-polar sites of the membranes. The interaction leads to the apparition of pores, causing disruption of the membrane and cellular rupture. These compounds have demonstrated antibacterial activity against bacteria, such as *P. aeruginosa*, *K. pneumoniane*, *S. typhi.* or *B. subtilis* [23]. However, in many cases, the mechanism of action is not yet understood. Lectins isolated from macroalga have gained attention due to their great range of bioactivities. *E. serra* and *G. marginata* lectins showed antibacterial activity against *V. vulnificus* and *V. pelagicus* through the interaction between these compounds and components of the bacterial cell wall, such as lipopolysaccharides or peptidoglycans [25]. Lectins extracted from *S. filiformis* presented inhibitory effects against Gram-negative bacteria, like *S. marcescens*, *S. typhi*, *K. pneumoniae*, *E. aerogenes*, *Proteus* sp. and *P. aeruginosa*. This effect was associated with the interaction between glycol-compounds present on the cell wall. In a similar way, lectins extracted from macroalga *H. musciformis* exhibited antifungal activity against *T. rubrum* and *C. lindemuthianum* [142]. They have also displayed antiviral effects against human immunodeficiency, hepatitis C, severe acute respiratory syndrome coronavirus (SARS-CoV) viruses, mainly by preventing the entry of the virus in the host cells and thereby their propagation [143].

The antimicrobial properties of macroalgae polysaccharides are attributed to the interaction between glyco-receptors of the bacterial cell wall, compounds of the membrane and nucleic acids and the polysaccharides. Those interactions lead to the disruption of the membrane stability and cellular functions [34]. Several factors have been shown to influence this activity, such as the molecular weight, charge density, structure and conformation [20]. Sulfated polysaccharides have demonstrated their antibacterial activity in several studies. For example, depolymerized fucoidans of *L. japonica* showed antibacterial activity against *E. coli* and *S. aureus*, which is caused by the interaction of fucoidans with membrane proteins, leading to membrane rupture and further cell death [137]. In other study, sulfated polysaccharides were extracted from different marine macroalgae and their antibacterial and antibiofilm properties were assessed against dental plaque bacteria. Fucoidan extracted from *F. vesiculosus* inhibited the mentioned bacteria and foodborne pathogens. In this case, the results suggest that fucoidan may not present a direct killing effect and may act by trapping nutrients, reducing the bioavailability [138]. To our knowledge, few studies have evaluated the antifungal properties and mechanisms of algal polysaccharides. Water soluble polysaccharides extracted from *P. capillacea* and *D. membranacea* displayed antifungal activity against different yeast and fungi. *P. capillacea* inhibited the growth of *F. oxysporium*, while *D. membranacea* inhibited *C. albicans* and *M. phaseli*. In future studies, it is expected that the compounds involved in this activity, as well as their mechanisms of action will be identified [140]. Regarding antiviral effects, these have been studied more extensively. Macroalgal polysaccharides can inhibit the multiplication of viruses such as the herpes simplex virus (HSV), human immunodeficiency virus (HIV) or the dengue virus. They can also obstruct the interaction between viruses and cells and inhibit enzymes [141]

The antibacterial activity of algal lipids and fatty acids has been attributed to their ability to inhibit the electron transport chain and oxidative phosphorylation in cell membranes, leading to the formation of peroxidation and auto-oxidation degradation products and the cellular lysis [34,145]. To our knowledge, no studies have isolated and then tested the antibacterial activity of macroalga fatty acids, but they have been successfully identified in bioactive extracts. In the study by El Shafay et al. [144], fatty acids were identified in the bioactive fraction of the extracts of *S. vulgare* and *S. fusiforme*, but no isolation was performed. The analysis demonstrated that the cell wall of *S. aureus* and *K. pneumoniae* was perforated, which resulted in the rupture of the cell wall, leakage of the cytoplasm and further cell death. Similarly, fatty acids were detected in the active fractions extracted from the red algae *G. edulis*. Although fatty acids were not isolated and their antimicrobial activity was not separately verified, authors attributed the antibacterial effects against *Vibrio* spp. and *A. hydrophila* to these compounds [145]. In the case of fungi, it has been proposed that fatty acids may act in disrupting the cell membrane, inhibiting the reproduction. Antifungal activity has been observed against *C. cladosporioides* and *C. sphaerospermum*, in addition to antiprotozoal effects against *T. cruzi* and *L. amazonensis* [147]. A sulfoquinovosyldiacylglycerol isolated from the *n-*BuOH fraction of *C. racemosa* showed antiviral effect against HSV type two by disturbing the early stage of the viral life cycle [148].

Among the literature, the most studied antimicrobial compounds are the polyphenols. Their antimicrobial action has been associated with their ability to alter membrane permeability (causing cell lysis), inhibit enzymes and different metabolic pathways, bind to surface molecules and other mechanisms. This activity seems to be related to the number of hydroxyl groups and also the degree of polymerization [20,23]. Several studies have assessed the antimicrobial properties and the diverse action mechanisms of polyphenols. For example, a recent study evaluated the antibactericidal action of phlorotannins (a type of polyphenol found in macroalgae of the class *Phaeophyta*) extracted from *F. vesiculosus*. The results showed that these compounds presented a significant bactericidal effect against *S. aureus*, *S. pneumonia* and *P. aeruginosa*. Phlorotannins presented a higher effectivity against Gram-positive bacteria than against Gram-negative bacteria, probably due the differences between their cell membranes, since Gram-negative bacteria are surrounded by an outer membrane which is rich in polysaccharides. The authors attributed the observed effects to the ability of phlorotannins to inhibit bacterial growth by the alteration of the cell membrane [129]. Similarly, phlorotannins from *S. thunbergii* have ben demonstrated to inhibit *V. parahaemolyticus*, causing damage to the cell wall and the membrane, which increased the membrane permeability and caused further leakage and destruction of the bacterial cells [130]. A phlorofucofuroeckol of the brown macroalga *E. bycliclis* has shown antibacterial effects against methicillin-resistant *S. aureus.* This compound produced damage in the cell membrane, leading to the leakage of cytoplasm and cell death. Furthermore, this compound suppressed the expression of genes related to resistance to methicillin in a dose-dependent manner [131]. Bromophenols, extracted from the red macroalga *K. alvarezii*, showed activity against *P. gingivalis*, the principal agent of chronic periodontitis. These compounds were able to downregulate the expression of the proteins involved in the infectious pathways of the bacteria [132]. Regarding yeast and fungi, the phlorotannin dieckol, extracted from *E. clava*, was tested against the fungi *T. rubrum*, associated with dermatophytic nail infections. The results exhibited alterations in membrane integrity and also in cell metabolism [133]. Another study evaluated the antifungal properties of the phlorotannins extracted from the brown macroalgae *C. nodicaulis*, *C. usneoides* and *F. spiralis* against different pathogenic yeast and fungi. Antifungal activity against all the studied species was observed, with the yeast *C. albicans* ATCC 10231 being the most susceptible, while the most susceptible fungi were *E. floccosum* and *T. rubrum.* The action mechanisms of phlorotannins were also evaluated. In the case of phlorotannins of *C. nodicalus* and *C. usneoides*, the results indicated a lower ergosterol content in the cell membrane of the yeast and fungi, respectively, which disrupted cellular integrity and functions. On the other hand, *F. spiralis* phlorotannins reduced the chitin content of the fungi cell wall, an essential wall component. Furthermore, they inhibited the formation of the germ tube of *C. albicans*, reducing its virulence and its capacity to adhere to epithelial cells. Finally, all phlorotannins increased the activity of the mitochondrial respiratory rate, which may increase the production of reactive oxygen species [134]. Phlorotannins also present antiviral activity. Five phlorotannins extracted from the brown algae *E. clava* displayed inhibitory effects against the Influenza A virus, through the inhibition of the neuraminidase, a critical enzyme for the life cycle of the virus [135]. Polyphenolic rich extracts from *E. arborea* and *S. filiformis* have demonstrated antiviral effects against Measles virus. Authors have proposed that polyphenols act by direct inactivation of the viral particle, which prevents the infection of cells [136].

Finally, in the case of pigments, the antimicrobial mechanism has not been fully understood. The most studied pigments are the carotenoids, which are supposed to act trough the accumulation of lysozyme, an enzyme able to digest bacterial cell walls [20]. Among carotenoids, fucoxanthin stands out and its antimicrobial properties have been tested against different pathogenic bacteria [34]. Recently, this compound has been tested against *E. coli*, *B. cereus*, *B. subtilis*, *K. pneumoniae*, *S. aureus*, *P. aeruginosa* and *L monocytogenes*. The proposed mechanisms consist of an increase in permeability, leakage of the cytoplasm and inhibition of nucleic acid formation [128]. Regarding antifungal activity of pigments, chlorophyll extracts from *S. pallidum* was tested against fungi *S. glycines* and *A. niger* and shown to possess low antifungal activity. However, the action mechanism has not been elucidated [150].

## 4. Novel Liquid–Liquid and Solid–Liquid Extraction Technologies to Efficiently Extract Algal Bioactive Compounds

Over the years, significant research efforts have been made to efficiently extract algae bioactive compounds by applying different methodologies. Conventional extraction methods (solid–liquid extraction) have numerous limitations, e.g., lower efficacy, high energy cost and low yield, thus new state-of-the-art extraction methodologies are required. Table 3 presents several articles in respect of the antimicrobial activity of macroalgae crude extracts, using the technologies mentioned below. 

### 4.1. Supercritical Fluid Extraction (SFE)

Supercritical fluid extraction (SFE) is a green analytical methodology used for the extraction of high-value bioactive compounds from complex matrixes [160]. SFE uses supercritical fluids, which above their critical point exhibit liquid-like characteristics such as solvent power, and negligible surface tension, as well as gas-like features such as enhanced transport properties.

Comparing SFE with other conventional extraction techniques, SFE presents several advantages, namely the use of minimal solvents, great extraction selectivity, short processing time, and a low degradability of the extract, showing a broad application for different bioactive compounds [161].

The thermodynamics properties of carbon dioxide (CO_2_) make it the preferred solvent for SFE-based extraction processes [151]. Moreover, due to its low toxicity, low cost, low explosivity, facile availability and environmentally friendly nature, it also presents major factors favoring the choice of CO_2_ as the SFE solvent [162]. Considering the physical characteristics, CO_2_ can only be used as the extraction solvent for the extraction of nonpolar or low polarity compounds (as supercritical CO_2_ is a nonpolar solvent). Nevertheless, the polarity of CO_2_ can be modulated using co-solvents such as small amounts of ethanol or methanol, increasing the extraction yields of polar compounds [162]. Recently, several reports described the application of SFE to extract high value bioactive molecules in arctic brown algae of the species *F. vesiculosus*. The arctic brown fraction extracts present a predominant content of fatty acids [151,163], polyphenols [163,164], carotenoids and chlorophylls [164]. Moreover, these artic brown algae SFE extracts also possess pronounced bacterial, fungicidal and immunostimulant activities [163.

Despite the potential of the SFE technique and its suitability to extract high-value bioactive compounds from algae, clearly the extraction depends on the nature of the target compounds. Using the SFE technique, a study concluded that *D. salina* extracts in the presence of SFE at 314 bar and 9.8 °C showed a substantial antimicrobial activity against *E. coli*, *S. aureus*, *C. albicans* and *A*. *niger*. As indicated in the work, this notable antimicrobial activity could be attributed to the presence of indolic compounds, PUFAs, and carotene metabolism, such as *β*-ionone and neophytadiene in the SFE *D. salina* extracts [151].

The antifungal potential of the brown algal *F. vesiculosus* was studied. To perform this study, algal extracts were obtained using SFE at a temperature of 50 °C and a pressure of 300 bar. Using aqueous algal extracts at the concentrations of 0.5% and 1.0%, a 100% growth inhibition of macroconidia within 144 h was obtained. Moreover, *F. vesiculosus* SFE extracts also promoted a 48% and 72% mycelial growth of phytopathogenic *F. oxy*sp*orum* and *F. culmorum*, respectively, after 168 h of incubation [152].

### 4.2. Ultrasound Assisted Extraction (UAE)

Ultrasound assisted extraction (UAE) technique uses acoustic waves in the kilohertz range (20 kHz to 100 kHz) that travel through the solvent producing cavitation bubbles. When the cavitation bubbles burst at the surface of the complex sample matrix, a shockwave induces damage to the sample cell wall enhancing the mass transfer of high-value bioactive compounds across cellular membranes into the solution [165]. Two different types of equipment can be used to carry out UAE: the ultrasonic bath (indirect sonication) that operates at a frequency between 40–50 kHz using a power of 50–500 W and the ultrasonic probe which operates only at 20 kHz. The principal difference between this equipment is the way that the ultrasound wave affects the sample [166]. 

The UAE technique is considered a cold extraction technique, as temperature during the extraction process is comparatively low and does not affect the stability of extracted compounds. UAE presents several advantages, such as the potential to reduce or eliminate the use of toxic chemical solvents and it is a more economic process (no need of supplementary energy to separate phases and to eliminate solvent). Moreover, using UAE, full extractions can be completed in a few minutes with high reproducibility, simplifying the manipulation and work-up, giving a higher purity to the final product and eliminating post-treatment of water waste [167].

UAE was the method of choice to extract bioactive compounds (total phenolics, fucose and uronic acid) from *A. nodosum.* To investigate the effect of process variables (extraction time, acid concentration, ultrasonic amplitude) response surface methodology (RSM) was employed. Higher extraction yields were obtained for total phenolics, fucose and uronic acid, respectively, at optimized extraction conditions of 25 min 0.03 M HCl and 114 μm of ultrasonic amplitude. Furthermore, it was demonstrated that UAE can be used to enhance the extraction of bioactive compounds from seaweed [168]. 

The extraction of phenolic compounds including gallic acid, catechins and their galloylated esters (gallates) in red and brown edible seaweeds, *Palmaria* sp., *Porphyra* sp., *H.elongata*, *L. ochroleuca* and *U. pinnatifida*, was carried out using ultrasonic bath, magnetic stirring and water bath with constant shaking [169].

UAE was also used to extract polysaccharides (fucose and glucans) from *L. digitata* [170] and *L. obtuse* [171]. In this case, the RSM was used to investigate the effect of the UAE variables (temperature, time and ultrasonic amplitude) on the macroalgal extracts to enhance the yields of polysaccharides and its antioxidant activities. A study observed that the UAE studied parameters showed significant influence on the levels of fucose. The highest fucose levels were obtained at optimized conditions of 76 °C during 10 min and ultrasonic amplitude of 100% using 0.1 M HCl as solvent [170]. While, the optimum UAE extraction parameters for the maximum phenolic content in *L. obtusa* extracts were a solvent seaweed ratio of 30:1; extraction temperature of 50 °C and extraction time of 42.8 min [171].

The same experimental design approach was used to compare UAE and microwave-assisted extraction (MAE), where the combination of both methodologies generated higher yields of compound extraction when compared to the use of UAE and MAE methods separately [172].

### 4.3. Microwave Assisted Extraction (MAE)

Microwave-assisted extraction (MAE) is one of the most advanced techniques used for the extraction of bioactive compounds from numerous seaweeds [173,174]. Microwaves are a nonionizing radiation with wavelengths ranging from as short as 1 mm to as long as 1 m and frequencies between 300 MHz and 300 GHz. Microwaves induce molecular motion in materials and solvents with dipoles, leading to subsequent heating of the sample. This heating leads plant cells to lose their moisture content through evaporation; the steam produced swells and ultimately ruptures the cells, releasing their bioactive components more easily. MAE of bioactive compounds might be affected by numerous factors, such as the frequency, power, time of extraction, moisture content and particle size of the sample, type and concentration of the solvent, ratio of solid to liquid, extraction temperature, extraction pressure, and number of extraction cycles [174,175].

Carrageenans from *S. chordalis* (Rhodophyceae,) harvested from the Brittany coast (France) were successfully extracted by MAE methodology. Native carrageenan extracted by MAE had the highest yield (29.3%) after 10 min at 90 °C. Evaluation of the antiviral activity of *S. chordalis* carrageenan against HSV-1 (Herpes simplex virus type one) showed a EC_50_ of the iota-carrageenans fractions in the range of 3.2 to 54.4 μg/mL (MOI 0.01 ID_50_/mL) with no cytotoxicity in that range of concentrations [176].

Microwave-assisted aqueous two-phase extraction was utilized for simultaneous extraction and separation of polysaccharides from *S. pallidum.* Using the optimal extraction conditions of 21% ethanol (w/w) and 22% ammonium sulfate (w/w), ratio of material to liquid 1:60 (g/mL), extraction time of 15 min, microwave power of 830 W, and extraction temperature 95 °C, an aqueous extracts rich in fucose, galactose, mannose, and glucuronic acid was obtained [177]. This approach demonstrated to be a high-efficient and practical method for the bioactive compounds extraction from seaweeds [178]. Others seaweeds such as *S. thunbergii*, and red algae *P. haitanensis* [179]; *G. lemaneiformis* [180]; *U. pertusa* [181]; *S. ceylonensis*, *U. lactuca*, *G. lemaneiformis* and *Durvillaea antarctica*, [180] were also subject to MAE to obtain polysaccharides. Shuntaro Tsubaki et al. [182] proved the efficacy of microwave-assisted hydrothermal extraction for the production of sulfated polysaccharides from *U. meridionalis*, *U. ohnoi* and *M. latissimum* [182].

Four seaweed species: *A. nodosum*, *L. japonica*, *L. trabeculata* and *L. nigrecens* were investigated for phenolic compounds extraction and their antioxidant capacity was also evaluated by MAE. These extracts presented a higher crude yield and higher total phenolic content when compared to conventional extraction techniques. MAE was also employed for the antioxidant extraction from green algae *Chaetomorpha* sp. [183]. Alternative microwave-assisted configurations such as microwave hydrodiffusion and gravity (MHG), were also used for the extraction of phenolic compounds in *L. ochroleuca* a brown seaweed [184]. 

Phlorotannin was obtained from *C. flexuosum*, *C. plumosum* and *E. radiata* by MAE. Using water as extraction solvent a most efficient extraction process with shorter processing times and a higher purity product was obtained [185]. The same compounds were also attained from the brown seaweed *C. sedoides* [186].

Pigments like fucoxanthin, were also recovered by MAE from *L. japonica*, *U. pinnatifida* and *S. fusiforme* [187]. MAE under optimum extraction conditions was an effective method to recover fucoidan from *F. vesiculosus*. [188] and from *E. radiata* [189].

Fucoidans from brown alga *N. zanardinii* [153] were isolated using conventional and non-conventional extraction procedures (subcritical water extraction, UAE and MAE), in order to evaluate the effects of the recent introduced technologies on the biochemical characteristics and saccharide composition of the extracts, along with their antibacterial, antiviral and cytotoxic properties. The highest and lowest fucoidan yields were obtained by sub critical water extraction and UAE, respectively. It has been reported that the use of different extraction methods resulted in the achievement of fucoidans with various chemical compositions and molecular weights. The algal extracts were tested against *E. coli*, *P. aeruginosa*, *L. monocytogenes* and *S. aureu*s. Fucoidans extracted by MAE and sub critical water extraction were able to inhibit the growth of *E. coli*. However, fucoidans isolated by EUAE, UAE and MAE showed inhibitory effects against *P. aeruginosa* [153].

A comparison between hot reflux extraction and MAE was conducted, leading to the conclusion that the amount of polysaccharides achieved by both techniques were similar [190]. Studies using hydrodistillation SFE and focused microwave-assisted hydrodistillation indicated that the highest extraction yield was obtained when SFE was used, even though the bioactive terpenes and fatty acids were obtained in greater quantity by the MAE method.

In other studies, MAE and UAE methodologies were shown to be the more efficient techniques for the chlorophyll and carotenoid isolation from freshwater green algae: *C. glomerata*, *C. rivularis* and *U. flexuosa* compared to the Soxhlet extraction and solid phase extraction [191].

The algal contents of *Oedogonium* sp., *Stigeoclonium* sp., *Ulothrix* sp. and *Nitzschia* sp. were extracted by MAE, using methanol an ethanol as extracting agents. The obtained crude extracts were tested against *E. coli*, *S. aureus* and *S. typhi*. All the four algal species inhibited Gram-positive and Gram-negative bacteria, except *S. aureus* which was resistant to the algal extracts. Only the ethanolic extracts of *Nitzschia* sp. and *Ulothrix* sp. showed antibacterial activity against this strain [154].

### 4.4. Enzymatic-Assisted Extractions (EAE)

Enzymatic-assisted extraction (EAE) is based on the innate ability that enzymes have to catalyze reactions with a high specificity, selectivity and an ability to function under mild processing conditions in aqueous solutions [192], the EAE of bioactive compounds from numerous sources, including marine ones, has received much attention in recent years. As compared to other reported conventional extraction methods, EAE offers some noteworthy advantages namely, high selectivity, overall efficacy, rapid extraction, eco-friendly procedures, low-energy consumption, minimal usage of harsh chemicals and process recyclability [160]. A range of enzymes including ligninolytic, cellulolytic and proteolytic enzymes has been extensively used as EAE catalysts. Enzyme-based pre-treatment or catalysis easily causes the breakdown and/or hydrolysis of complex materials on the cell walls and membranes, thus also supporting the recovery of intracellular bioactive constituents which are not easily extractable through conventional extraction methods [193].

A study used an EAE coupled with UAE to achieve the eco-friendly extraction of soluble bioactive fractions from the macroalga *S. muticum*. Using a mixture of commercial complex enzyme formulations, these authors obtained an *S. muticum* extract rich in bioactive components such as carbohydrates, monosaccharides, oligosacaccharides and polyphenols [194]. A similar strategy was used by other authors [195] to extract bioactive compounds from green and brown seaweed *C. tomentosum*, *S. muticum* and *O. pinnatifida.* Extracts obtained from those seaweeds were evaluated for proximate characterization and biological properties. According to the authors, high extraction yields of cellulase, viscozyme, nitrogen, total phenolics and sugars and sulfated polysaccharide were measured. 

EAE was used (using five carbohydrases and three proteases) to extract multiple bioactive compounds such as polyphenols, proteins and polysaccharides from several seaweeds, namely *S. boveanum*, *S. angustifolium*, *P. gymno*sp*ora*, *C. cervicornis*, *C. sinuosa*, *I. stellata* and *F. irregularis* [155]. Several fractions of the seaweed extracts were used against *S. aureus*, *E. faecalis*, *S. cerevisiae*, *B. cereus* and *A. hydrophila.* Although antimicrobial activities of the enzymatic extracts were low, flavourzyme resulted in a higher number of seaweed extracts with antimicrobial activity against foodborne pathogens [155].

### 4.5. Accelerated Solvent Extraction (ASE)

Accelerated solvent extraction (ASE) also referred as pressurized liquid extraction (PLE) is deemed an excellent technique for the extraction of polar compounds [196,197]. ASE is a solid–liquid extraction process performed at high temperatures (50–200 °C) and high pressures (10–15 MPa). Its main advantages over traditional extraction methods are the dramatic decrease in the amount of solvent used and extraction time [198].

A recent study used ASE to extract phenolic contents (using water, ethanol:water and acetone:water as extraction solvents) from algae *A. nodosum*, *P. canaliculata*, *F*. *spiralis* and *U. intestinalis* [199]. This paper compared ASE with the conventional solid–liquid extraction technique. It was observed that ASE was more effective for the extraction of polyphenols when acetone:water (80:20) was used as solvent. However, the traditional solid–liquid extraction (using ethanol:water (80:20) or 100% of water) resulted in higher phenolic content in brown macroalgal extracts.

Another experiment used ethyl acetate and water by ASE to extract bioactive compounds from *P. pavonica* seaweed. High-performance thin-layer chromatography was used to analyze the ethyl acetate and water ASE *P. pavonica* extracts. Chromatograms indicated that several families of compounds (terpenes, flavonoids and amino acids) were extracted from the studied seaweed but only the ethyl acetate extracts contained polyphenols and lipids [200].

In another case, the antimicrobial activity of *H. elongata* ASE extracts was assessed. In this work, the use of several solvents (ethanol, hexane, and water) demonstrated that ASE technology was able to extract a variety of bioactive molecules namely, fatty acids, tocopherols, alkanes and phenols from *H. elongata*. The highest yield was reached when only water was used as the extraction solvent. Additionally, the antibiotic and antifungal activity were also tested in *S. aureus*, *E. coli* and fungus *C. albicans* and *A. niger*. In this case, the best results were obtained when ethanol was used as the ASE solvent [158].

ASE was also used to extract several bioactive compounds such as phlorotannins, phosphatidylcholine, betaine lipids, chlorophylls and carotenoids from the algae *S. muticum* [201,202]. A study used a combination of ASE and water extraction to withdrawal molecules of interest from seaweeds. Furthermore, those authors [159] also tested the antimicrobial activity of *F. vesiculosus* extracts against *E. faecium*, *MRSA*, *K. pneumoniae*, *A. baumannii*, *P. aeruginosa*, *E. coli*, *C. albicans*, *C. neoformans*, *V. anguillarum*, *P. bacteriolytica* and *P. elyakovii.* Although *F. vesiculosus* extracts produced evident MRSA growth inhibition, radical scavenging and pro-apoptotic activities, this study highlighted the significant effect of seasonal sampling on these activities [159].

### 4.6. Intensity Pulsed Electric Fields (IPEF)

In intensity pulsed electric fields (IPEF), the complex biological samples are placed between two electrodes hosted in a treatment chamber and exposed to high intensity electric fields (10–50 kV/cm), applied in the form of repetitive pulses of very short duration (from several nanoseconds to a few milliseconds), inducing the permeabilization of the cell membranes by electroporation, easing the subsequent release of intracellular matter as demonstrated in a recent study with microalgae [203,204]. The advantages of IPEF are short treatment time, low treatment temperature, increase in shelf life, increased extractive yield, use of both batch and continuous processes and improved metabolite extraction [203].

Polikovsky and co-workers [205] investigated a new technology to process macroalgae into biorefinery, employing efficient energy and zero waste conversion of macroalgae biomass into food, chemicals and fuels. For this purpose, a selective extraction of proteins from *Ulva* genus green macroalgae was carried out using an IPEF process. Using 75 pulses with an average electric field strength of 3 kV/cm and pulse duration of 6 μs, several proteins were extracted from the *Ulva* genus, namely, calreticulin, ferredoxin-NADP^+^ reductase, fructose-1,6-bisphosphatase, fructose-bisphosphate aldolase 1, phosphoglycerate kinase and ribosomal protein L12 (chloroplast). Polikovsky demonstrated that IPEF process, despite some proteins were partially or completely degraded by the pulse electric field treatment, this method is selective and efficient [205].

## 5. Applications

### 5.1. Food Industry and Animal Feed

Nowadays, it has been demonstrated that synthetic antimicrobials, like sodium benzoate, sodium nitrite or sorbic acid, used in the food industry, can produce negative effects on the consumer’s health. Thus, the search for new antimicrobials, obtained from natural sources is gaining importance [206]. Different studies have reported the inhibitory effects of macroalgae compounds against food-borne pathogens such as *E. coli*, *L. monocytogenes*, *S. aureus* or *Salmonella* sp. Antimicrobials could be included in both the food products as novel functional ingredients, or in the packaging material [207]. In the first case, antimicrobials could be added to the food directly to prevent the spoilage due to the growth of microorganisms. Recently, the polysaccharide fucoidan extracted from *F. vesiculosus* showed in vitro inhibitory effects against *L. monocytogenes* and *S. enterica* serovar Typhimurium. Furthermore, this compound was included in the formulation of a functional apple beverage. The results showed that fucoidan was significantly effective in preventing the spoilage produced by the mentioned pathogens [208]. On the other hand, antibacterial compounds isolated from macroalgae could be used in the active packaging sector, which faces the same spoilage produced by microorganisms. Studies in this area have been focused on the incorporation of antimicrobial compounds into the packaging material to eliminate or reduce the growth of pathogens, extending the shelf life of the products. Currently, several edible films contain alginates and carrageenans to prevent microbial spoilage. For example, carrageenan and chitosan containing films demonstrated inhibitory effects against *C. jejuni* and lactic acid bacteria in raw chicken breast. Biodegradable films containing *F. spiralis* extract and sorbic acid have been developed to enhance the shelf life of food products [34].

Regarding animal feed, antimicrobial compounds like laminarin and fucoidan could be used in the animal feeding to substitute commercial antibiotics (which may cause negative effects both on animals treated and environment), enhance the animal survival and also the safety and nutritional characteristics of animal products consumed by humans [209]. For example, the administration of fucoidan to monogastric animals inhibited the attachment of bacterial species in the gut and also obstructed the binding of *Enterococci* and *Streptococci* sp. to the extracellular matrix, preventing the colonization of the mucosa [209]. Likewise, fucoidan and other antimicrobial compounds are useful tools in aquaculture, which can increase the resistance of the aquatic species against bacterial and viral diseases. For instance, dietary supplementation with fucoidan, alginate and ulvan have demonstrated the ability to improve the survival of several fish and crustaceans species against bacteria and viruses that threaten aquaculture production [210].

### 5.2. Cosmetics and Pharmaceuticals 

Considering the large amount of studies that have demonstrated that some compounds in macroalgae might have antimicrobial capabilities, there is a possibility that these compounds may be used in the development of treatments against pathogens and diseases. In Table 4, some examples of the antimicrobial properties against bacteria, antibiotic-resistant bacteria, fungi and viruses have been shown. For example, polyphenols could be used in dental products for the treatment of chronic periodontitis [132], nail treatments to eliminate fungi [134] or in the development of new antivirals [135,136], among other interesting applications. Polysaccharides have shown antimicrobial effects against bacteria and fungi, but their antiviral properties are gaining attention in the pharmaceutical sector [132]. In the case of other compounds, like proteins and pigments, their antimicrobial properties have not yet been widely studied and understood, so their current pharmaceutical applications are limited. Regarding cosmetic applications, several macroalgal compounds could be interesting to develop safe treatments against acne. A β-D-galactosyl O-linked glycolipid has been identified as the main compound responsible of the inhibitory effects of the ethyl acetate extract of *F. evanescens* against *C. acnes.* This bacterium can colonize the skin and is related with acne. Finally, the phenolic compound fucofuroeckol-A, isolated from methanolic extract of *E. bicyclis* also showed antimicrobial properties against the mentioned microorganism [33]. Nevertheless, most of antimicrobial studies are in vitro and thus, to ensure the efficacy and safety of these compounds, further in vivo studies are required.

### 5.3. Anti-Fouling

Surfaces immersed in the marine environment could be colonized by biofouling organisms, such as bacteria, protist or invertebrates [226]. Several paints containing toxic compounds like tributyltin, mercury or arsenic have been designed to eliminate these communities. However, these paints contribute to marine contamination, affecting the food chain and producing genetic mutations [227]. Therefore, the development of alternative solutions is of great importance. Antimicrobial compounds from macroalgae could be used for this purpose [33]. Although most of studies have tested different extracts, some of them have identified the compounds involved in the antifouling properties [226]. Several of these compounds have been identified in green, brown and red macroalgae such as pigments, polyphenols or fatty acids. For example, polyphenolic compounds from the green algae *U. pertusa* showed anti-algal properties against red tide microalgae [228]. Phlorotannins from *Sargassum* sp. are allelochemicals, which several studies have demonstrated to be exuded by the macroalgae into the surrounding water to prevent the settlement of epiphytes in their surface [229]. Finally, some antifouling compounds have been identified in the red algae *L. translucida*, mainly fatty acid derivatives [230]. In the following years, an increase in the identification of compounds with antifouling activities is expected, as well as the development of their biotechnological applications. 

Other interesting approach to obtain anti-fouling products is the inhibition of the quorum sensing of bacteria, defined as a cell-to-cell communication system, based on the production, release of and perception of molecules by the bacterial cells. These molecules are involved in a great variety of processes, including biofilm formation [227]. Several extracts and compounds derived from macroalga with the ability to inhibit quorum sensing have been reported. For example, Carvalho et al. (2017) [231], reported that *C. cervicornis* extracts inhibited the growth of *C. violaceum*, due to the inhibition of the quorum sensing, since no killing effect was observed. A study conducted with *P. gymnospora* demonstrated that the *a*lpha-bisabolol obtained from this alga inhibited significantly the quorum sensing and biofilm formation of *S. marcescens* [232]. Recently, several new α-pyridones, derived from *E. prolifera* have been identified. The results of the gar diffusion method showed that four of these compounds significantly inhibited the expression of genes involved in the quorum sensing in *P. aeruginosa* [233]. Further research on macroalgal extracts and compounds could be promising to develop new applications, as a strategy to reduce the use of antimicrobials [232]. 

## 6. New Approaches and Future Perspectives 

In the previous sections and throughout the manuscript a series of applications of the antimicrobial compounds obtained from algae is given. Among all these applications, the most common is the use of these molecules or extracts as preservatives of natural origin in the food, cosmetic or pharmaceutical industry. Namely, in the food industry new trends are rapidly being incorporated to the market such as active packaging or the use of antibiofilm compounds [14,34]. Additionally, research on natural antimicrobials has also increased due to the current problem related to resistant strains. In this regard, polyphenols represent molecules widely employed by in vitro studies for their synergistic effect when acting with other conventional antimicrobials and thus, resulting in a higher efficacy, lower doses and side-effects reduction [234]. However, to our knowledge, few studies have assessed the in vivo properties of polyphenols or other enriched extracts obtained from algae. 

On the other hand, nanoparticles (NPs) and nanomaterials have recently gained popularity both in engineering and medical sciences. Algae have been proposed as alternative nanobiofactories for the synthesis of NPs since physicochemical synthesis is usually difficult and expensive. Algae can facilitate metal reduction as they can accumulate metal ions and stabilize and remodel them. Likewise, algae extracts are known for their biological properties and particularly, they have been studied for their antibacterial and antifungal effects [235]. Their mechanism of action consists basically of the NPs adhesion to the microorganism surface and their penetration into cells. Once there, different interactions can occur with the bacterial components: disruption of the enzymatic activity or the production of reactive oxygen species (ROS) that can result in mitochondrial, protein or DNA damage [236]. The use of algae in NPs synthesis is quite a new trend and therefore, different authors have performed studies on characterizing, synthetizing and researching their antimicrobial potential. Table 4 shows a series of examples of algae-based NPs and their antimicrobial activity. For instance, a study found that silver NPs based on four different algae (*U. faciata*, *P. capillacae*, *J. rubens* and *C. sinusa*) could reduce the bacterial growth of *S. aureus* and *E. coli* on textile cotton fabrics. This capacity could be useful for the design of antiseptic dressings with biomedical applications [217]. In another case, also Ag-based NPs obtained from the red algae *G. amansii*, showed potential as antifouling for their antimicrobial activity against different microorganisms [212]. Among all the examples given on Table 4, the majority of the studies have been carried out on silver-NPs, however other approaches are being investigated too and their antimicrobial activity has been proved against plenty of microorganisms. Moreover, other approaches apart from antimicrobial or antifouling applications have been proposed, such as their use in bioremediation [237]. On the other hand, the use of algae extracts as prebiotics has also been studied. Marine algae have been pointed out as a source of oligosaccharides and polysaccharides and even though these compounds possess several bioactivities, their use as prebiotics is rising because of their high content of dietary fiber, enhancing the growth of those beneficial bacteria in the intestinal tract [11,238]. 

Taken together, all the information given in the different manuscript sections and considering the favorable consumer perception about these products, there is a huge potential antimicrobials, extracted from algae, to be incorporated into different applications. Future perspectives should focus on the development of further in vivo and toxicological studies and in the transformation of the whole process into a cost-effective and reproducible alternative which goes through the improvement, further study and optimization of the extraction techniques of the bioactive compounds responsible for the antimicrobial activity [239,240].

## 7. Conclusions 

The use of bioactive compounds as a constituent in several food–cosmetic- and medical-based products will soon be the norm. Among different sources, macroalgae biomass as a marine by-product has been considered as a promising and viable source of a broader range of bioactive compounds. As evident from this review, algae bio-products include: proteins, peptides, polysaccharides, polyphenols, fatty acids, pigments, and the highest treasure antimicrobial compounds. To obtain these valuable bioactive compounds, several innovative green extraction methodologies have been used to efficiently recover them from algae biomass. This review also comprises the recent advancements in the extraction techniques such as supercritical-fluid extraction, accelerated solvent extraction, ultrasound-, microwave-, and enzymatic-assisted extraction. Most of the literature concerning the antimicrobial activity of algae is based on classic extraction techniques. Furthermore, new future approaches should focus on the utilization of natural antimicrobial compounds obtained from algae into the pharmaceutical industry, due to the current problem related to the resistance of the drugs to pathogenic microorganisms. Moreover, algae have been also implicated as a possible alternative for nanoparticles and nanomaterials synthesis, which will raise the possibility to use this by-product in engineering and medical science.

## Figures and Tables

**Table 1:** Antimicrobial activity of algae crude extracts obtained using classic extraction technologies from 2010 to 2020.

Alga Species	Conditions	Bacteria Tested	Main Outcomes	Ref.
*L. brandenii*—(India)	HAE, MeOH: CHCl_3_ (6:4), 35 °C, 120 rpm, 7 days	*S. aureus/B. subtilis/M.* *luteus/R. rhodochrous/E. coli/P.* *aeruginosa/Vibrio cholerae/Salmonella typhi/Streptococcus pneumoniae*	All organisms were inhibited. High activity against *B. subtilis* whereas it was moderate against *E. coli*	[35]
*G. ornata*—(Brazil)	HAE, H_2_O, 25 °C, 24 h	*B. subtilis/S. aureus/E. aerogens/E. coli/P. aeruginosa/S. choleraesuis/S. typhi*	Only exhibits inhibition to *E. coli*	[36]
*C. rubrum*—(Chile)	HAE (3x), 96% EtOH, 24 h.	*S. parasitica/Y. ruckeri*	Antibacterial and antifungal activities against fish pathogens	[37]
*G. changii*—(Malaysia)	Solid liquid extraction, MeOH, 4 days.	*P. aeruginosa*	Minimal inhibitory concentration (MIC) 6. 25 mg/mL	[38]
*P. capillacea/O. obtusiloba*—(Brazil)	Solid liquid extraction, cold Hx and 70% EtOH.	*E. coli/S. aureus/Salmonella* sp*./V. harveyi*	No antimicrobial activity.	[39]
*P. gymno*sp*ora*—(Brazil)	Percolation with MeOH	*S. aureus*	MIC 500 µg/mL	[40]
*S. latifolium/S. platycarpum/C. socialis*—(Arabian Gulf)	HAE extraction, MeOH and AcO 25 °C, 150 rpm, 7 days	*S. aureus/S. xylosus/MRSA/E. faecalis/B. subtilis/E. coli/P. aeruginosa/Salmonella* sp.*/K. pneumoniae/C. albicans*	Higher activity against Gram positive bacteria than Gram negative	[41]
*L. japonica*—(Korea, Japan, China)	HAE (x3), EtOH, 25 °C, 1 day.	*S. mutans/S. sobrinus/A. naeslundii/A. odontolyticus/A. actinomycetemcomitas/F. nucleatum/P.gingivalis*	Inhibitory activity against all microorganisms	[42]
*D. membranacea—*(Mediterranean Sea)	Column extraction, EtOH, AcO and MeOH/DCM	*S. aureus/S. agalactiae/B. subtilis/E. faecium/E. faecalis/E. coli/C. albicans*	EtOH and AcO showed higher antimicrobial activity	[43]
*S. oligocystum*—(Persian Gulf)	HAE, hot and cold H_2_O and glycerin	*S. aureus/S. epidermidis/P. aeruginosa/E. coli*	Hot water extract exhibited activity against *S. aureus*, *S. epidermidis*, and *P. aeruginosa*	[44]
*C. myrica/C. trinodis/P. gymno*sp*ora/S. dentifolium/S. hystrix/A. fragilis/C. racemosa/C. fragile*—(Red sea)	HAE, MeOH, 25 °C, 50 rpm, 7 days.	*E. coli/S. aureus/E. faecalis/Salmonella* sp./*B. cereus/P. aeruginosa.*	MeOH extracts *P. gymno*sp*ora* and *C. fragile* showed the highest activities	[45]
*S. polycystum/P. australis*—(Malasya)	HAE, Hx, DCM, MeOH, 72 h	*S. aureus/B. cereus/E. coli/E. coli/P. aeruginosa*	*S. polycystum* extracts exhibited higher bacteriostatic activity	[46]
*Gracilaria* sp.—(Malaysia farmed algae)	HAE, MeOH, 48 h	*B. subtilis/S. aureus/S. epidermidis/E. coli/V. cholera/E. cloacae.*	Moderate antibacterial activity but *S. aureus*, *S epidermidis*, *E. cloacae* were not inhibited	[47]
*P. antillarum/P. boergesenii/U. flexuosa—*(Persian Gulf)	HAE, EtOAc, MeOH; 48 h	*B. subtilis/B. pumulis/E. faecalis/S. aureus/S. epidermidis/E. coli/K. pneumoniae/P. aeruginosa/A. niger/C. albicans/S. cerevisiae*	*A. niger*, *P. aeruginosa* were not inhibited*. P. antillarum* extracts do not have effect in *K. pneumoniae*	[48]
*U. lactuca/U. intestinales/C. vagabunda—*(Black sea)	HAE, EtOH 70%	*S. aureus/E. coli*	Antibacterial activity was higher in *E. coli*	[49]
*U. rigida—*(Turkey)	HAE, DCM; then DCM/MeOH	*S. agalactiae./S. aureus./E. faecalis/Micrococcus* sp./*V. tapetis/V. anguillarum/V. alginolyticus E. coli/P. cepacia/P. fluorescens/P. aeruginosa/A. salmonicida/A. hydrophila/S. typhimurium*	No significant variation with seasons. The most sensitive bacteria were *A. salmonicida*, *S. typhimurium*, *S. agalactiae*, *A. hydrophila*, *P. cepacia*, *S. aureus* and *E. faecalis*	[50]
*G. doryphora—*(Egypt)	HAE, MeOH, EtOH, EtOAc, 72 h, 150 rpm.	*B. subtilis/E. faecalis/S. aureus/E. coli/P. aeruginosa/C. albicans*	Inhibitory effects except against *E. coli*	[51]
*E. prolifera/U. reticulata/C. myrica/P. pavonica/T. triquetra/S. portieriatum/G. multipartita—*(Saudi Arabia)	HAE, PeEt, DEt, EtOAc, MeOH, 30 °C, 120 rpm, 24 h	*B. subtilis/MRSA/S aureus/E. coli/K. pneumoniae/P. aeruginosa*	*P. pavonica* and *T. triquetra* extracts showed better activity. In some cases, inhibitory effects changed with seasons	[52]
*G. multipartita/U. reticulata/S. marginatum—*(Turkey)	HAE, Hx, DCM, MeOH, 72 h	*B. subtilis/MRSA/S. aureus/E. coli/K. pneumoniae/P. aeruginosa*	*B. subtilis*, *MRSA*, and *E. coli* were susceptible	[53]
*L. obtusa/C. elongatum/C. multifida*—(Adriact sea)	Soxhlet extraction, AcO	*B. mycoides/B. subtilis/S. aureus/E. coli/K. pneumoniae/A. flavus/A. fumigatus/C. albicans/P. verrucosum*	All the tested extracts showed antimicrobial activity	[54]
*C. iyengarii/*S. *a*sp*erum/S. marginatum/C. indica/S. variegatum/S. swartzii/S. tenerrimum/S. ilicifolium/I. stellata/S. robusta/H. tuna/R. implexum/D. dichotoma var. intricata/D. indica/M. afaqhusainii/J. laminarioides—*(Pakistan)	HAE, EtOH, 1 week	*B. subtilis/S. aureus/E. coli/S. typhi/P. aeruginosa/R. solani/Macrophomina phaseolina/F. solani/F. oxysporum*	Brown species have shown more potential than red algal species. The highest antibacterial activity was found in EtOH extract of *D. dichotoma* var *intricata* and *D. indica* against *S. typhimurium*	[55]
*C. linum*/*C. rupestris*/*G. dura*/*G. gracilis*/*G. longissima*/*U. prolifera*—(Italy)	Soxhlet extraction, CHCl_3_/MeOH, 60 °C, 24 h	*V. ordalii/V. salmonicida V. alginolyticus/V. splendidus/V. harveyi/V. vulnificus*	Different susceptibilities to algal extracts were detected. *G. longissima* was the most effective	[56]
*C. rupestris*—(Mediterranean Sea)	HAE, MeOH, CHCl_3_, H_2_O	*Enterococcus* sp.*/S. agalactiae/V. cholerae*	Results showed seasonal variety	[57]
*G. longissima*—(Mediterranean Sea)	Soxhlet extraction, CHCl_3_/MeOH (2:1), 60 °C, 24 h	*P. aeruginosa/Enterococcus* sp.*/S. agalactiae/V. salmonicida/V. fluvialis/V. vulnificus/V. cholerae/V. alginolyticus/C. albicans/C. famata/C. glabrata*	Moderate antimicrobial effect except on *V. salmonicida* and fungal species	[58]
*C. antemmina/C. peltata/C. scalpelliformis/D. dichotoma/S. marginatum/A.* sp*ecifera/G. lithophilia/G. corticata*—(India)	HAE, MeOH	*E. coli/P. aeruginosa/S. aureus/K. pneumoniae*	*G. lithophila* presents the most promising results	[59]
*J. rubens/C. elongata/P. capillacea/U. fasciata/U. lactuca/E. compressa/E. linza/S. vulgare/C. sinuosa*—(Egypt)	HAE, EtOH 70%, MeOH 70% AcO 70%, 150 rpm, 72 h	*B. subtilis/S. aureus/E. coli/S. typhi/K. pneumoniae/C. albicans.*	In all the tests, AcO showed the biggest inhibition halos	[60]
*D. flabellata/P. concrescens/L. johnstonii/G. martinensis/U. lactuca/C. fragile*—(Mexico)	HAE, AcO:MeOH	*E. coli/S. aureus/B. cereus/B. subtilis/S. epidermidis*	*L. johnstonii*, *D. flabellata* and *U. lactuca* presented activity against pathogenic bacteria tested	[61]
*E. bicyclis*—(South Korea)	HAE, MeOH, 70 °C, 3 h	*C. acnes/S. aureus/S. epidermidis/P. aeruginosa*	Inhibitory effects except against *P. aeruginosa*	[62]
*C. trinodis*—(Persian Gulf)—(Persian Gulf)	HAE, DEt:EtOH:Hx	*S. aureus/S. epidermidis/E. coli/P. aeruginosa*	*The best* Inhibitory effect was against *S. epidermidis* was the worst against *P. aeruginosa*	[63]
*C. glomerata,/E. linza/U. rigida/C. barbata/P. pavonica/C. ciliatum/C. officinalis—*(Black sea Turkey)	HAE, 95% EtOH	*S. aureus/B. cereus/A. niger/S typhimurium/L. monocytogenes/E. coli/C. albicans/P. aeruginosa*	All alga extracts present antimicrobial activity	[64]
*S. vulgare/C. hirsutus/R. verruculosa—*(Coast of Algeria)	Soxhlet extraction, MeOH, MeOH: CHCl_3_, 6h	*B. cereus/S. aureus/M. luteus/P. aeruginosa/E. coli/K. pneumoniae/C. albicans*	Positive antimicrobial results against *S. aureus* and *B. cereus*	[65]
*Laurencia* ssp. (*aldingensis/catarinensis/dendroidea/intricata/translucida*) –(Brazil)	HAE, Hx, CHCl_3,_ MeOH, H_2_O	*C. albicans/C. parapsilosis/C. neoformans*	*L. aldingensis* showed the best antifungal effects	[66]
*D. membranacea*—(Tunisia)	HAE, H_2_O, CHCl_3,_ EtOAc	*S. aureus/S. epidermidis/L. monocytogenes/M. luteus/E. faecium/E. coli/P. aeruginosa/S. typhimurium/C. albicans/C. kefyr/C. krusei/C. dubliniensis/C. glabrata*	Inhibitory effects against *M. luteus*, *S. aureus*, *S. epidermidis*, *L. monocytogenes*, C*. krusei*, *C. dubliniensis and C. kefyr*	[67]
*S. wightii/C. linum/P. gymno*sp*ora*—(India)	HAE, Hx, EtOAc, AcO, MeOH	*P. aeruginosa/S. typhi/E. amylovora/E. aerogens/P. vulgaris/K. pneumonia/E. coli/MRSA/B. subtilis/E. faecalis*	EtOAc and AcO extracts were more efficient, but no inhibitory effects were observed against *S. paratyphi* and *K. pneumonia.*	[68]
*Fucus* spp*./P. elongata/Rhodomela confervoides/S. latissima./C. rupestris/D. contorta/F. vesiculosus/C. rubrum/M. stellatus/L. digitata*—(Germany)	HAE, DCM	*E. amylovora/E. coli/P. aeruginosa/B. subtilis/S. lentus*	The macroalgae presented antibacterial activity against at least one of the test strains	[69]
*H. tuna/C. barbata/C. bursa*—(Montenegro)	HAE, DCM:MeOH, 48 h	*E. coli/S. aureus/B. subtilis/E. faecalis/C. albicans*	*C. barbata* demonstrated as having the best antimicrobial activity for *S. aureus* and *B. subtilis*	[70]
*G. corticata/G. edulis*—(India)	HAE, DMSO, 70% MeOH, 130 rpm, 16 h	*E. coli/Photobacterium* sp./*P. fluorescens/S. aureus/B. subtilis*	MeOH and DMSO extracts inhibited *B. subtilis*	[71]
*L. digitata/S. latissima/H. elongata/P. palmata/C. cri*sp*us*—(Ireland)	HAE, MeOH, EtOH, AcO, 2 h	*L. monocytogenes/S. abony/E. faecalis/P. aeruginosa*	The extraction of antimicrobials from macroalgae were solvent dependent	[72]
*S. marginatum*—(India)	HAE, DCM, EtOAc, AcO, MeOH	*Candida* spp.	Low antifungal properties.	[73]
*S. lomentaria/P. pavonica/C.mediterranea/H. musciformis/*S. *filamentosa*—(Turkey)	HAE, MeOH, 8 h, 200 rpm	*S. aureus/S. typhimurium/E. coli/E. faecalis/C. albicans*	*S. lomentaria* inhibited *S. typhimurium*. *C. mediterrranea* inhibited *C. albicans*	[74]
*U. lactuca/E. intestinalis—*(Adriatic coast of Montenegro)	HAE, Hx, DCM, MeOH, 72 h	*B. mycoides/B. subtilis/E. coli/K. pneumoniae/S. aureus/A. flavus/A. fumigatus/C. albicans/P. purpurascens/P. verrucosum*	Inhibitory effects were observed against *B. mycoides* and *B. subtilis*	[75]
*A. fragilis/C.a myrica/H. cuneiformes/L. papillosa/S. cinereum/T turbinata—*(Egypt)	HAE, 80% MeOH, 25 °C	*B. subtilis/S. aureus/E. coli/C. albicans*	*H. cuneiformis* extract showed stronger activity	[76]
*E.cava—*(Korea)	HAE, EtOH, *n*-Hx, DCM, EtOAc, *n*-BuOH, H_2_O	*S. aureus/MRSA/S. typhi/S. enteritidis/S. gallinarum*	EtOH had antibacterial activity *S. aureus*, *MRSA* and *Salmonella* spp.	[77]
*C. barbata*—(Red Sea, Egypt)	Soxhlet extraction, EtOH	*B. subtilis/S. aureus/M. luteus/E. coli/P. aeruginosa/Serratia. marcescens/S. typhi/Vibrio* sp./*A. hydrophila/C. albicans*	Inhibitory activity except against *M. luteus*	[78]
*K. alvarezii*—(Malaysia)	HAE, EtOH, H_2_O	*E. coli/B. cereus*	*B. cereus* was inhibited but no *E. coli.*	[79]
*U. lactuca/D. dichotoma/P. gymno*sp*ora/S. vulgare/H. musciformis/D. simplex*—(Brazil)	HAE, DCM, MeOH, EtOH, H_2_O	*T. rubrum/T. tonsurans/T. mentagrophytes/M. canis/M. gypseum/E. flocossum/C. albicans/C. krusei/C. guilliermondi/C. parapsilosis/*	EtOH and MeOH extracts were the most effective	[80]
*B. bifurcata*—(Portugal)	HAE, MeOH, DCM, 12 h	*E. coli/P. aeruginosa/B. subtilis/S. aureus/S. cerevisiae*	MeOH extracts had inhibitory effects in all the microorganisms	[81]
*H. flagelliformis/C. myrica/S. boveanum*—(Persian Gulf)	HAE, DCM, 48 h	*E. coli:/K. pneumonia/S. typhi/S. aureus/S. epidemidis/B. subtilis/A. niger/C. albicans*	The antimicrobial activity was solvent-dependent	[82]
*T. conoides*—(India)	HAE, ***n***-Hx, MeOH and EtOH: H_2_O, 72 h.	*S. aureus/S. epidermidis/E. coli/P. aeruginosa/A. niger/C. albicans*	MeOH and EtOH: H_2_O extracts were the most effective against the microorganisms studied	[83]
*D. dichotoma/P. pavonica/S. vulgare*—(Adriatic Sea)	HAE, AcO, 50 °C;4 h	*B. mycoides/B. subtilis/S. aureus/E. coli/K. pneumoniae/A. flavus/A. fumigatus/C. albicans/P. purpurescens/P. verrucosum*	All crude extracts have a statistically significant inhibitory effect on microbial growth	[84]
*C. racemosa/C. sertularioides/K. alvarezii*—(Malaysian coast)	HAE, Hx, CHCl_3_, EtOAc, EtOH, MeOH, H_2_O, 1 day	*B. cereus/S. aureus/A. baumannii/E. coli/K. pneumoniae/P. aeruginosa/C. albicans/C. parapsilosis/C. krusei/C. neoformans/A. fumigatus/T. interdigitale*	Inhibitory effects except against A. *fumigatus*	[85]
*U. lactuca*—(Gulf of Maine)	HAE, MeOH, 70 °C	*S. aureus/S. epidermidis*	Inhibitory effects against both species	[86]
*T. ornata/T. decurrens/T. conoides/S. polycystum/S. incisifolium/S. ilicifolium/H.a cuneiformis*—(Madagascar)	HAE, MeOH, EtOAc	*B. cereus/S. aureus/S. pneumoniae/E. cloacae/K. oxytoca/S. boydii/E. coli/S. enteridis/P. aeruginosa/C. albicans/C. membranaefaciens/C. neoformans/T. mucoides*	Antimicrobial tests of the crude extracts revealed a strong activity against *S. aureus* and *S. pneumoniae*	[87]
*A.* sp*ecifera/Cladophoropsis* sp.*/L. paniculata/Tydemania* sp.*/U. prolifer*a	Soxhlet extraction, EtOH and PeEt, 24 h	*C. albicans/A. niger/Mucor* sp.*/Paeciliomyces* sp.	EtOH extract of *L. paniculata* showed the best antimicrobial activity	[88]
*H. e*sp*eri/C. prolifera—*(Egypt)	Soxhlet extraction, MeOH, 40 °C, 24 h	*E. coli/P. aeruginosa/S. typhimurium/A. hydrophila/B. subtilis/S. aureus*	Inhibitory effects against *B. subtilis* and *S. aureu*s growth but no against *P. aeruginosa* and *S. typhimurium*,	[89]
*Grateloupia* sp./*G. corticata/Halymenia* sp./*Metamastophora* sp./Sp*yridia* sp.	HAE, MeOH, 24 h	*E. cloacae/K. oxytoca/E. coli/S. enteridis/B. cereus/S. aureus/S. pneumoniae/C. albicans.*	All the crude extracts obtained can inhibit microbe’s growth.	[90]
*H. elongata*—(Ireland)	HAE, H_2_O, MeOH, 40 °C, 100 rpm, 2 h	*L. monocytogenes/S. abony/E. faecalis/P. aeruginosa*	60% MeOH extract showed the best results.	[91]
*F. serratus/F. vesiculosus*—(Ireland)	HAE, H_2_O, MeOH, EtOAc, AcO	*MRSA 28 strains*	Both species present antibacterial activity against several MRSA strains.	[92]
*U. reticulata*—(Vietnam)	HAE, MeOH:CHCl_3_: H_2_O	*B. cereus/S. faecalis/E. cloace/S. aureus/E. coli/P. aeruginosa/V. haveyi*	*U. reticulata* showed high antimicrobial activity, against *E. cloace* and against *E. coli*.	[93]
*U. rigida*—(Tunisia)	HAE, EtOH:H_2_O, 48	*B. subtilis/B. cerus/S. aureus/S. epidermis/E. faecalis/L. monocytogenes/E. coli/P. aeruginosa/K. pneumoniae/A. niger/F. graminearum/F. culmorum/F. oxy*sp*orum/C. albicans*	Antimicrobial activity varied depending on the season	[94]
*U. fasciata/G. salicornia—*(Honolulu, USA)	HAE, EtOH	*E. faecalis/V. alginolyticus/V. cholerae/S. aureus/S. typhimurium/E. coli*	*U. fasciata* had significantly higher antimicrobial activity compared to *G. salicornia*	[95]
*U. lactuca/D. dichotoma/C. elongata*—(Algeria)	HAE, MeOH, DEt, CHCl_3_	*E. coli/S. aureus/Salmonella/C. albicans/Penicillium* sp.	CHCl_3_ extracts of *U. lactuca* and *C. elongata* had the highest activity against *E. coli* and *Salmonella* sp. MeOH of all species showed antifungal activity for *C. albicans*.	[96]

**Conditions:** Heat-assisted extraction (HAE) Acetone (AcO), Ethanol (EtOH), Methanol (MeOH); Dichloromethane (DCM), Water (H_2_O), Hexane (Hex), Dimethilsulfoxide (DMSO), Chloroform (CHCl_3_), Petroleum ether (PeEt), Ethyl acetatete (EtOAc), Diethyl ether (DEt), *n*- Butanol (*n*-BuOH), *n*-Hexane (*n*-Hx). **Main outcomes**: Minimal inhibitory concentration (MIC).

**Table 2:** Antimicrobial properties of compounds extracted from macroalgae.

Type	Compounds	Macroalgae	Antimicrobial Activity	Ref.
Polyphenols	Phlorotannins	*F. vesiculosus*	Alteration of the cell membrane and cell destruction of *S. aureus*, *S*. *pneumonia* and *P. aeruginosa*	[129]
	Phlorotannins	*S. thunbergii*	Alteration of the cell membrane, cytoplasm’s leakage and cell destruction of *V. parahaemolyticus*	[130]
	Phlorofucofuroeckol	*E. bicyclis*	Cell membrane damage and suppression of genes related to methicillin resistance in *S. aureus*	[131]
	Bromophenols	*K. alvarezii*	Downregulation of pathogenic genes of *P. gingivalis*	[132]
	Dieckol	*E. clava*	Alteration of cell integrity and metabolism of *T. rubrum*	[133]
	Phlorotannins	*C. nodicaulis*, *C. usneoides*, *F. spiralis*	Alterations of the cell wall composition, increased mitochondrial respiration. Inhibition of the formation of the germ tube of *C. albicans*	[134]
	Phlorotannins	*E. clava*	Inhibition of the enzyme neuraminidase of the Influenza A virus	[135]
	Polyphenolic rich extracts	*E. arborea*, *S. filiformis*	Inhibition of the viral particle	[136]
Polysaccharides	Depolymerized fucoidans	*L. japonica*	Interaction with protein of the cell membrane and cellular rupture of *E. coli* and S*. aureus*	[137]
	Fucoidan	*F. vesiculosus*	Inhibition of dental plaque bacteria and foodborne pathogens.	[138]
	Laminarin rich extracts	*A. nodosum*, *L. hyperborea*	Inhibition of *S. aureus*, *L. monocytogenes*, *E. coli* and *S. typhimurium*.	[139]
	Water soluble polysaccharide extracts	*P. capillacae*, *D. membranacea*	Inhibition of *F. oxy*sp*orium* Inhibition of *C. albicans* and *M. phaseli*	[140]
	Sulfated polysaccharides	*G. skottbergii*	Obstruction of herpes simplex virus type 1 and 2 attachment to the cells	[141]
			Interference with fusion between HIV infected cells. Inhibition of the viral enzyme reverse transcriptase	
		*C. okamuranus*	Inhibition of dengue virus by interaction with the glycoprotein of the viral envelop	
Proteins & peptides	Lectins	*E. serra*, *G. marginata*	Inhibition of the growth of *V. vulnificus* and *V. pelagicus*. Interaction between lectins and components of the bacterial cell wall	[25]
	Protein hydrolysate fraction	*S. longicruris*	Inhibition of *S. aureus* growth	[105]
	Lectins	*S. filiformis*	Inhibition of several Gram-negative bacteria by interaction with compounds of the cell wall	[142]
	Lectins	*H. musciformis*	Inhibition of *T. rubrum* and *C. lindemuthianum*	
	Lectins	*B. coacta*, *Griffithsia* sp.	Antiviral effects against HIV, Hepatitis C virus and SARS-CoV by preventing the entry in the host cells	[143]
Fatty acids	Bioactive fraction	*S. vulgare*, *S. fusiforme*	Perforation of the cell wall of *S. aureus* and *K. neumoniae*, cytoplasmic leakage and cell death	[144]
	Bioactive fraction	*G. edulis*	Rupture of cell membrane of *Vibrio* spp and *A*. *hydrophila*	[145]
	Bioactive fraction	*S. marginatum*, *U. lactuca*	Fatty acids could be involved in the inhibition *S. aureus*, *E. coli* and *P. vulgaris*	[146]
	Bioactive fraction	*B. tenella*	Inhibition of *C. cladosporioides* and *C.* s*phaerospermum* by disrupting the cell membrane	[147]
	Sulfoquinovosyldia- cylglycerol	*C. racemosa*	Antiviral effects against HSV type 2 by disturbing the initial stages of the viral life cycle	[148]
Pigments	Fucoxanthin	*H. elongata*	Inhibition of *L. monocytogenes*	[149]
	Fucoxanthin	*Commercial extract*	Inhibition of several pathogenic bacteria by increasing cell membrane permeability, leakage of cytoplasm and inhibition of nucleic acid	[128]

**Table 3:** Antimicrobial activity of macroalgae crude extracts obtained using emerging extraction technologies.

Method	Conditions	Macroalgae	Bioactive Compound	Main Outcomes	Ref.
SFE	314 bar, 10 °C	*D. salina*	Fucoidans	Inhibition of *E. coli*, *S. aureus*, and *C. albicans*, growth	[151]
	300 bar, 50 °C	*F. vesiculosus*	Fucosterol	Inhibition of *Fusarium* sp.	[152]
UAE	200 W, 20 kHz; 55 °C; 20 min	*N. zanardinii*	Fucoidans	No activity against *E. coli*, *L. monocytogenes P. aeruginosa* and *S. aureus*	[153]
MAE	1500 W; 150 °C; 10 min (× 2)	*N. zanardinii*	Fucoidans	Positive activity against *E. coli*	[153]
	50 °C, 500 W, 10 min, MeOH, EtOH	*Oedogonium* sp./*Stigeoclonium* sp./*Ulothrix* sp./*Nitzschia* sp.	n.d.	All extracts inhibited at least one microorganism tested	[154]
UMAE	65 °C, 3 h (x 2)	*N. zanardinii*	Fucoidans	Inhibition of *P. aeruginosa*, but no effect on *E. coli L. monocytogenes* and *S. aureus*	[153]
EAE	Alcalase: 2.5 mL (2.4 U/g), pH 8, 50 °C, 24 h Flavourzyme: 2.5 mL (500 U/g), pH 7, 50 °C, 24 h Cellulase: 2.5 g (3 U/mg), pH 4.5, 50 °C, 24 h Viscozyme: 2.5 mL 100 fungal β-glucanase U/mL, pH 4.5, 50 °C, 24 h	*N. zanardinii*	Fucoidans	No activity against *E. coli L. monocytogenes*, *P. aeruginosa* and *S. aureus*	[153]
	Viscozyme L: pH 4.5 (0.1M, AB), 50 °C; AMG 300 L: pH 4.5 (0.1M, AB), 60 °C; Celluclast: pH 4.5 (0.1M, AB), 50 °C; Termamyl: pH 6 (0.1 M, SPB), 60 °C; Ultraflo: pH 6 (0.1 M, SPB), 40 °C; Flavourzyme: pH 7 (0.1 M, SPB), 50 °C; Alcalase: pH 8 (0.1 M, SPB), 50 °C; Neutrase: pH 8 (0.1 M, SPB), 50 °C;	*S. boveanum*, *S. angustifolium*, *P. gymno*sp*ora*, *C. cervicornis*, *C. sinuosa*, *I. stellate*, *F. irregularis*	Polyphenols/ polysaccharides	*F. irregularis* extracts obtained with Viscozyme, Celluclas and Flavourzyme inhibited *S. aureus. P. gymno*sp*ora* and *C. sinuosa* extracts obtained with Celluclast inhibited E. feacalis.	[155]
EUAE	65 °C, 3 h (× 2)	*N. zanardinii*	Fucoidans	Inhibition of *P. aeruginosa,* but no effect on *E. coli L. monocytogenes* and *S. aureus*	[153]
SWE	1500 W, 150 °C, 10 min (× 2)	*N. zanardinii*	Fucoidans	Inhibition of *E. coli* and *P. aeruginosa*, but no effect on *L. monocytogenes* and *S. aureus*	[153]
SWH	200–280 °C, 1.3–6.0 MPa, Catalyst -Acetic acid	*L. japonica*	n.d.	Strong antibacterial activity against *S. typhimurium* and *E. coli*,	[156]
PLE	Hx, EtOAc, AcO, EtOH, EtOH: H_2_O (50:50)	*U. intestinalis*, *U. lactuca*, *F. vesiculosus*, *D. dichotoma*, *C. baccata*, *H. elongata*	Fatty acids	*F. vesiculosus* extract exhibited the best antimicrobial properties	[157]
	Hx, EtOH, W; 200 °C, 20 min	H. *elongata*	Fatty acids/pigments	All extracts presented antimicrobial activity against *S. aureus*, *E. coli*, *C. albicans* and *A. niger*	[158]
	H_2_O, MeOH, DCM Temperature (20, 40, 60 °C)	*F. vesiculosus*	Phlorotannins/phosphatidylcholine/ betaine/ lipids/chlorophylls/carotenoids	*F. vesiculos* extract was only effective as an antimicrobial agent to MRSA	[159]

**Conditions**: Acetone (AcO), Ethanol (EtOH), Methanol (MeOH); Dichloromethane (DCM), Water (H_2_O), Hexane (Hex), Ethyl acetatete (EtOAc). n.d: not described. **Methods**: Supercritical fluid extraction (SFE), Ultrasound Assisted Extraction (UAE), Microwave-assisted extraction (MAE), Ultrasonic-Microwave Assisted Extraction (UMAE), Enzymatic-Assisted Extraction (EAE), Enzymatic-Ultrasound Assisted Extraction (EUAE), Subcritical Water Extraction (SWE), Subcritical Water Hydrolysate (SWH), Pressurized Liquid Extraction (PLE).

**Table 4:** Macroalgae-based nanoparticles (NPs) with antimicrobial properties.

Macroalgae	NPs	Size (nm)	Antimicrobial Activity	Ref.
*S. muticum*	Ag	43–79	Growth inhibition of *B. subtilis*, *K. pneumoniae* and *S. typhi*	[211]
*G. amansii*	Ag	27–54	Antifouling activity against *P. aeruginosa*, *V. parahaemolyticus*, *E. coli*, *A. hydrophila*, *B. pumilus* and *S. aureus*	[212]
*G. corneum*	Ag	20–50	Antimicrobial and antibiofilm activity against *C. albicans* and *E. coli*	[22]
*G. corticata*	Ag	18–46	Antifungal activity against *Candida* spp.	[213]
*G. birdiae*	Ag	20–95	Antimicrobial activity against *E. coli*	[214]
*S. wightii*	Ag	55–70	Maximum growth inhibition against *M. luteus* > *S. marcescens*	[215]
*V. pachynema*	Ag	30–40	Moderate growth inhibition against *M. luteus* > *S. marcescens*	[215]
*P. hornemannii*	Ag	70–75	Antimicrobial activity against the fish pathogens: *V. parahaemolyticus*, *V. vulnificus*, *V. harveyii* and *V. anguillarum*	[216]
*U. faciata*	Ag	7	Bacterial reduction in textile fabrics against *S. aureus* and *E. coli*	[217]
*P. capillacea*	Ag	7	Bacterial reduction in textile fabrics against *S. aureus* and *E. coli*	[217]
*J. rubens*	Ag	12	Bacterial reduction in textile fabrics against *S. aureus* and *E. coli*	[217]
*C. sinusa*	Ag	20	Bacterial reduction in textile fabrics against *S. aureus* and *E. coli*	[217]
*S. plagiophyllum*	AgCl	18–42	Growth inhibition of *E.a coli*	[218]
*S. marginatum*	Au	18–94	Growth inhibition of *P. aeruginosa*, *K. oxytoca*, *E. faecalis*, *K. pneumoniae*, *V. parahaemolyticus*, *V. cholerae*, *S. typhi*, *S. paratyphi*, and *P. vulgaris*	[219]
*S. plagiophyllum*	Au	65–66	Antibacterial activity against *S. typhi* and *E. coli* by bacteria membrane lysis	[220]
*C. sinuosa*	Fe_3_O_4_	11–34	Excellent antifungal activity against *A. flavus* and *F. oxysporum.* Antibacterial activity against *E. coli*, *P. aeruginosa*, *S. typhi*, *V. cholera*, *B. subtilis* and *S. aureus*	[221]
*P. capillacea*	Fe_3_O_4_	16–23	Antibacterial activity against *E. coli*, *P. aeruginosa*, *S. typhi*, *V. cholera*, *B. subtilis* and *S. aureus*	[221]
*U. flexuosa*	Fe_3_O_4_	12	Antibacterial activity against *B. subtilis*, *S. aureus*, *E. coli*, *E. faecalis* and *S.s epidermidis*	[222]
*S. wightii*	ZrO_2_	4.8	Enhancement of the antibacterial activity against *B. subtilis*, *E. coli* and *S. typhi*	[223]
*S. wightii*	MgO	68	Antibacterial activity against *S. pneumonia*, *MRSA 11*, *MRSA 56*, *E. coli*, *P. aeruginosa* and *A. baumannii*	[224]
*S. myriocystum*	ZnO	76–186	Growth inhibition against Gram (+) bacteria: *S. mutans* > *M. luteus* and Gram (-): *Neisseria gonorrohea* > *V. cholera* > *K. pneumonia.* Antifungal activity against *C. albicans > A*. *niger*	[225]

## Abbreviations

***Generic***	
ATCC	American type culture collection
DW	Dry weight
FW	Fresh weight
HVS	Herpes simplex virus
MIC	Minimal inhibition concentration
MRSA	Methicillin-resistant *Staphylococcus aureus*
MOI	Multiplicity of infection
NPs	Nanoparticles
RSM	Response surface methodology
RT	Room temperature
SARS	Severe acute respiratory syndrome
UV	Ultraviolet
***Extraction techniques***	
ASE	Accelerated solvent extraction
EAE	Enzyme-assisted extraction
EUAE	Enzymatic ultrasound assisted extraction
HAE	Heat-assisted extraction
IPEF	Intensity pulsed electric fields
MAE	Microwave-assisted extraction
PLE	Pressurized liquid extraction
SFE	Supercritical fluid extraction
UAE	Ultrasound assisted extraction
***Compounds***	
DEt	Diethyl ether
DCM	Dichloromethane
DPPH	1,1-diphenyl-2-picryl hydrazyl
EtOAc	Ethyl acetate
EtOH	Ethanol
H_2_O	Water
Hx	Hexane
MeOH	Methanol
*n*-BuOH	*n*- Butanol
*n*-Hx	*n*-Hexane
PUFAs	Poly-unsaturated fatty acids
***Bacteria species***	
***Acinetobacter (A.):** A. baumannii. **Actinobacillus (A.):** A. actinomycetemcomitas. **Actinomyces (A.):** A. naeslundii, A. odontolyticus. **Aeromonas (A.):** A. hydrophyla, A. salmonicida. **Aspergillus (A.):** A. flavus, A. fumigatus, A. niger. **Bacillus (B.):** B. cereus, B. mycoides, B. pumilus, B. subtilis. **Candida (C.):** C. albicans, C. dublinensis, C. famata, C. glabrata, C. guilliermondi, C. kefyr, C. krusei, C. membranaefaciens, C. parapsilosis. **Campylobacter (C.):** C. jejuni. **Chromobacterium (C.):** C. violaceum. **Colletotrichum (C.):** C. lindemuthianum. **Cryptococcus (C.):** C. neoformans. **Cultibacterium (C.):** C. acnes. **Enterobacter (E.):** E. aerogens, E. cloacae. **Enterococcus (E.):** E. faecalis, E. faecium. **Epidermophyton (E.):** E. flocossum. **Erwinia (E.):** E. amylovora. **Escherichia (E.):** E. coli. **Fusarium (F.):** F. graminearum, F. culmorum, F. oxysporum, F. solani. **Fusobacterium (F.):** F. nucleatum. **Klebsiella (K.):** K. oxytoca, K. pneumonia. **Lactobacillus (L.):** L. brevis. **Listeria (L.):** L. innocua, L. monocytogenes. **Leishmania (L.):** L. amazonensis. **Macrophomina (M.):** M. phaseolina. **Methicillin-resistant Staphylococcus aureus** (MRSA). **Micrococcus (M.):** M. luteus**. Microsporum (M.):** M. canis, M. gypseum. **Penicillium (P.):** P. verrucosum. **Porphyromonas (P.):** P. gingivalis**. Pseudoalteromonas (P.):** P. bacteriolytica. **Proteus (P.):** P. vulgaris. **Pseudomonas (P.):** P. aeruginosa, P. cepacia, P. fluorescens. **Rhizoctonia (R.):** R. solani. **Rhodococcus (R.):** R. rhodochrous. **Saccharomyces (S.):** S. cerevisiae. **Salmonella (S.):** S. abony, S. choleraesuis, S. gallinarum, S. typhi, S. typhimurium. **Septoria (S.):** S. glycines**. Serratia (S.):** S. marcescens. **Shigella (S.):** S. boydii. **Staphylococcus (S.):** S. aureus, S. enterica, S. epidermidis, S. lentus, S. xylosus. **Streptococcus (S.):** S. agalactiae, S. epidermis, S. mutans, S. pneumonia, S. pyogenes, S. sobrinus. **Streptomyces (S.):** S. purpurascens. **Syspastospora (S.):** S. parasitica. **Trichophyton (T.**): T. interdigitale, T. mentagrophytes, T. rubrum, T. tonsurans, **Trichosporon (T.):** T. mucoides. **Trypanosoma (T.):** T. cruzi. **Vibrio (V.):** V. alginolyticus, V. anguillarum, V. cholerae, V. fluvialis, V. haryevi, V. ordalii, V. parahaemolyticus, V. salmonicida, V. splendidus, V. tapetis, V. vulnificus. **Yersinia (Y.)**: Y. ruckeri.*	
***Macroalgae species***	
**Chlorophyta** ***: Boodlea (B.):** B. coacta**. Caulerpa (C.):** C. peltata, C. prolifera, C. racemosa**,** C. scalpelliformis, C. sertularioides. **Cladophora (C.):** C. glomerata, C. rivularis, C. rupestris, C. socialis, C. vagabunda. **Codium (C.):** C. bursa, C. elongatum, C. fragile, C. iyengarii, C. tomentosum. **Enteromorpha (E.):** E. linza, E. prolifera. **Halimeda (H.):** H. tuna. **Monostroma (M.):** M. latissimum. **Rhizoclonium (R.):** R. implexum. **Ulva (U.):** U. fasciata, U. flexuosa, U. intestinalis, U. lactuca, U. meridionalis, U. ohnoi, U. pertusa, U. prolifera, U. rigida, U. reticulata. **Valonopsis (V.):** V. pachynema.* **Rhodophyta** *: **Acanthophora (A.):** A. specifera. **Actinotrichia (A.):** A. fragilis. **Ascophyllum (A.):** A. nodosum. **Ceramium (C.):** C. ciliatum, C. rubrum. **Chaetomorpha (C.):** C. antemmina, C. linum. **Chondrus (C.):** C. crispus. **Corallina (C.):** C. elongata, C. officinalis. **Digenea (D.):** D. simplex. **Dumontia (D.):** D. contorta. **Eucheuma (E.):** E. serra. **Galaxaura (G.):** G. cylindriea, G. marginata. **Gelidium (G.):** G. amansii, G. corneum. **Gigartina (G.):** G. skottsbergii. **Gracilaria (G.)**: G. birdiae, G. changii, G. corticata, G. dura, G. edulis, G. gracilis, G. lemaneiformis, G. multipartita, G. ornata, G. vermiculophylla. **Gracilariopsis (G.):** G. longissima. **Grateloupia (G.):** G. doryphora, G. lithophilia. **Hypnea (H.):** H. esperi, H. flagelliformis, H. musciformis. **Jania (J.):** J. rubens. **Kappaphycus (K.):** K. alvarezii. **Laurencia (L.):** L. aldingensis, L. brandenii, L. catarinensis, L. dendroidea, L. intricata, L. johnstonii, L. obtusa, L. paniculata, L. papillosa, L. translucida. Mastocarpus (M.): M. stellatus. **Melanothamnus (M.):** M. afaqhusainii. **Osmundaria (O.):** O. obtusiloba. **Osmundea (O.):** O. pinnatifida. **Palmaria (P.):** P. palmata. **Polysiphonia (P.):** P. elongata. **Porphyra (P.):** P. dioica, P. haitanensis. **Portieria (P.):** P. hornemannii. **Pterocladia (P.):** P. capillacea**. Pterocladiella (P.):** P. capillacea. **Rissoella (R.):** R. verruculosa. **Rhodomela (R.):** R. confervoides. **Sarcodia (S.):** S. ceylonensis.* **Ochrophyta*: Bifurcaria (B.):*** *B. bifurcata. **Canistrocarpus (C.):** C. cervicornis. **Cladosiphon (C.):** C. okamuranus. **Cladostephus (C.):** C. hirsutus. **Carpophyllum (C.):** C. flexuosum, C. plumosum. **Colpomenia (C.)**: C. sinuosa. **Cutleria (C.):** C. multifida. **Cystoseira (C.):** C. barbata, C. indica, C. mediterranea, C. myrica, C. nodicaulis, C. sedoides, C. trinodis, C. usneoides. **Dictyopteris (D.):** D. membranacea. **Dictyota (D.):** D. dichotoma, D. flabellata, D. indica. **Dilophus (D.):** D. fasciola. **Ecklonia (E.):** E. arborea, E. cava, E. kurome, E. radiata. **Eisenia (E.):** E. bicyclis. **Feldmannia (F.):** F. irregularis. **Fucus (F.):** F. evanescens, F. serratus, F. spiralis, F. vesiculosus. **Gymnogongrus(G.):** G. martinensis. **Himanthalia (H.):** H. elongata. **Hormophysa (H.):** H. cuneiformes. **Iyengaria (I.):** I. stellata. **Jolyna (J.):** J. laminarioides. **Laminaria (L.):** L. digitata, L. hyperborea, L. japonica. **Lessonia (L.):** L. nigrecens, L. trabeculata. **Nizamuddinia (N.):** N. zanardinii. **Padina (P.):** P. australis, P. concrescens, P. gymnospora. **Pelvetia (P.):** P. canaliculata. **Porolithon (P.):** P. antillarum, P. boergesenii. **Saccharina (S.):** S. latissima, S. longicruris. **Sargassum (S.):** S. angustifolium, S. boveanum, S. cinereum, S. dentifolium, S. hystrix, S. ilicifolium, S. incisifolium, S. latifolium, S. marginatum, S. muticum, S. myriocystum, S. oligocystum, S. pallidum, S. platycarpum, S. polycystum, S. portieriatum, S. swartzii, S. tenerrimum, S. thunbergii, S. variegatum, S. vulgare, S. wightii. **Scytosiphon (S.):** S. lomentaria. **Solieria (S.):** S. chordalis, S. filiformis, S. robusta. **Spatoglossum (S.):** S. asperum. **Spyridia (S.):** S. filamentosa. **Stoechospermum (S.):** S. marginatum. **Taonia (T.):** T. atomaria. **Turbinaria (T.):** T. conoides, T. decurrens, T. ornata, T. triquetra. **Undaria (U.):** U. pinnafitida.*	
